# Bioinformatic Discovery of a Cambialistic Monooxygenase

**DOI:** 10.1021/jacs.3c12131

**Published:** 2024-01-10

**Authors:** Chang Liu, Magan M. Powell, Guodong Rao, R. David Britt, Jonathan Rittle

**Affiliations:** † Department of Chemistry, University of California, Berkeley, Berkeley, California 94720, United States; ‡ Department of Chemistry, University of California, Davis, Davis, California 95616, United States

## Abstract

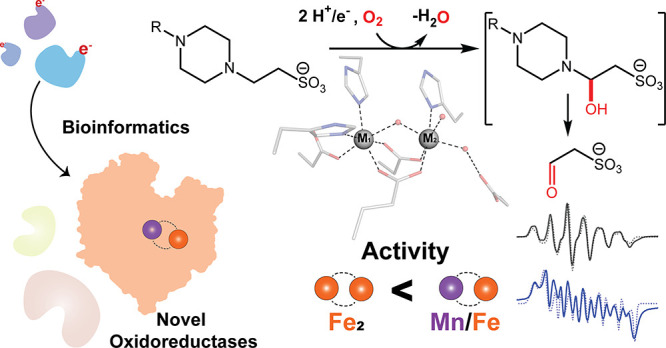

Dinuclear monooxygenases mediate challenging C–H bond oxidation reactions throughout nature. Many of these enzymes are presumed to exclusively utilize diiron cofactors. Herein we report the bioinformatic discovery of an orphan dinuclear monooxygenase that preferentially utilizes a heterobimetallic manganese–iron (Mn/Fe) cofactor to mediate an O_2_-dependent C–H bond hydroxylation reaction. Unlike the structurally similar Mn/Fe-dependent monooxygenase AibH2, the diiron form of this enzyme (SfbO) exhibits a nascent enzymatic activity. This behavior raises the possibility that many other dinuclear monooxygenases may be endowed with the capacity to harness cofactors with a variable metal content.

Most metalloenzymes harness a specific metallocofactor to perform their biological functions. Prototypical non-heme monooxygenases, such as Rieske oxygenases or soluble methane monooxygenase, are generally thought to require iron cofactors.^1,2^ In rare instances, the elemental content of a metallocofactor can be perturbed with the maintenance of nascent biological activity. These so-called cambialistic enzymes offer organismal resilience in the face of changing elemental bioavailability^3,4^ and provide a unique opportunity to understand redox tuning by proteins. However, examples of cambialistic redox enzymes are presently limited to certain superoxide dismutases^5−7^ and ring-cleaving dioxygenases^8^ that can function with either iron- or manganese-containing cofactors.

Recently, we demonstrated that AibH1H2 is a Mn/Fe-dependent monooxygenase that hydroxylates an aliphatic C–H bond of 2-aminoisobutyric acid.^9^ The protein components of AibH1H2 stem from a largely uncharacterized structural superfamily, PF04909, containing over 100,000 unique proteins that can individually function as either carboxylases, hydratases, hydrolases, or monooxygenases.^10−15^ Representatives of the latter enzyme class—collectively defined here as “amidohydrolase-related dinuclear oxygenases” (AROs)—include PtmU3^13^ and AibH2,^9,15^ which employ dinuclear cofactors with different metal identities. In contrast, the other characterized PF04909 enzymes either lack a cofactor^10^ or employ a mononuclear metal site that can be Mn or Zn.^11,16^ The primary structural features that differentiate mono- versus dinuclear active sites, engender monooxygenase functionality, and control the metalation of AibH2 and PtmU3 are unknown.

Since very few AROs have been described, we sought a means for their *in silico* identification. We first considered that the genes encoding AibH1H2 lie within an operon containing a small Rieske protein (AibG) predicted to bind a [2Fe–2S] cluster ligated by the canonical amino acid motif (**C**-*x-***H**-*x*_*n*_*-***C-***x*_2_-**H**).^15,17^ It was speculated that AibG serves as an endogenous reductase to AibH1H2 akin to other multicomponent monooxygenases (e.g., cytochrome P450, sMMO; Figure 1A)^1,18^ that require interprotein transfer of electrons for the corresponding monooxygenation reaction. This observation informs our bioinformatic hypothesis that many redox-active PF04909 proteins (e.g., AROs) could be identified by the inspection of neighboring sequences that encode reductase proteins contained within the same transcriptional unit. Herein we utilize this “guilt by association” strategy to identify four candidate AROs that can each harbor redox-active dinuclear active sites. One of these proteins was found to initiate an O_2_-dependent dealkylation reaction involving homolytic C–H bond cleavage. Remarkably, this reaction was found to proceed in protein samples that contain either an embedded Fe_2_ or a Mn/Fe cofactor, with a preference for the latter.

**Figure 1:**
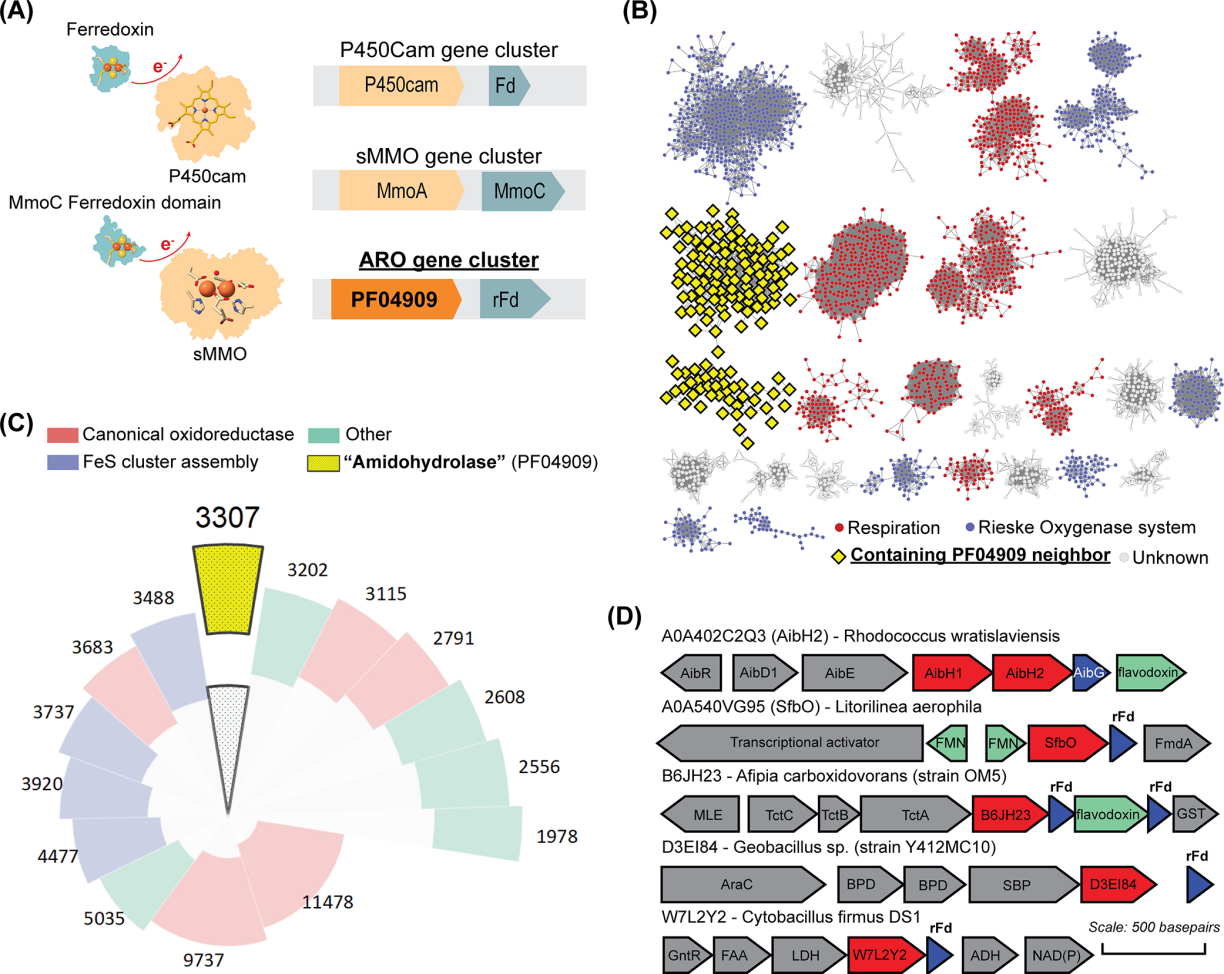
(A) Bioinformatic strategy to identify new AROs. (B) SSN of the PF00355 protein family (<250 amino acids) drawn at e-values <10^–30^ and colored according to the inferred function of the Rieske protein (Supporting Information). (C) Ranked abundance of PF00355 neighbor genes (Table S1). (D) Genome neighborhood diagrams of AibH2, SfbO, and the candidate AROs described. rFd refers to Rieske ferredoxin.

To identify new AROs, we prepared a sequence similarity network (SSN)^19^ of the Rieske protein superfamily PF00355 and evaluated the genomic neighborhoods of small Rieske proteins (<250 amino acids) to identify homologues of AibG. Many of the resultant SSN clusters (Figure 1B) harbor sets of Rieske proteins whose biological function can be inferred on the basis of their surrounding genes. The coding sequences for these proteins were often found adjacent to those of prototypical Rieske dioxygenases,^17^ respiratory cytochromes,^20^ or components of the biosynthetic iron sulfur cluster (Suf) machinery.^21^ However, these prototypical redox partners were absent in the genomic neighborhoods of a handful of SSN clusters that instead contain representative PF04909 sequences within three coding regions. In fact, these “amidohydrolases” represent the ninth most abundant protein family neighboring the analyzed Rieske proteins (Figure 1C). Accordingly, we hypothesize that these widespread Rieske proteins are AibG homologues and that their adjacent “amidohydrolases” are uncharacterized AROs which collectively form the basis for multicomponent monooxygenase systems.

To validate these bioinformatic predictions and establish the structure–function relationships of the emergent ARO family, we characterized a handful of representative proteins from a diverse taxonomic range of microorganisms. The genomic neighborhoods (Figure 1D) of candidate AROs stemming from *Litorilinea aerophila* (A0A540VG95, referred to as SfbO), *Afipia carboxidovorans* (B6JH23), *Cytobacillus firmus* (W7L2Y2), and *Geobacillus* strain Y412MC10 (D3EI84) intimate disparate physiological functions. Each of these proteins was fused to an N-terminal His_6_ tag, recombinantly expressed in *Escherichia coli*, and purified as soluble protein preparations (see the Supporting Information). For initial studies, the expression medium was supplemented with additional Fe^II^ sources to ensure the formation of an intact dinuclear active site. A 1.37 Å structure of SfbO (Figure 2A, PDB entry 8SM6) obtained on a single crystal grown under aerobic conditions confirms the presence of an Fe_2_ cofactor ligated by two *cis*-oriented (μ,κ^1^,κ^1^)-carboxylates, one κ^1^-carboxylate, and three κ^1^-histidine ligands derived from the protein side chains. One bridging μ-OH_*x*_ ligand was found between the two metal ions, and two terminal aquo ligands were observed on the Site 2 metal. One of these aquo ligands forms strong hydrogen-bonding interactions with an active site SO_4_^2–^ ion. This ion appears to be tightly bound via strong electrostatic and hydrogen-bonding interactions with ^67^N, ^232^R, ^235^R, ^280^Y, and ^341^W, and this observation may provide insight into functional groups present on the natural substrate (*vide infra*). Excepting the sulfate-binding interactions found in SfbO, the active site structure closely mimics that found in AibH2,^9^ PtmU3^13^ (Figure S2), and the other Fe_2_-metalated ARO proteins characterized herein (Figure S1, PDB entries 8SM7, 8SM8, and 8SM9).

**Figure 2:**
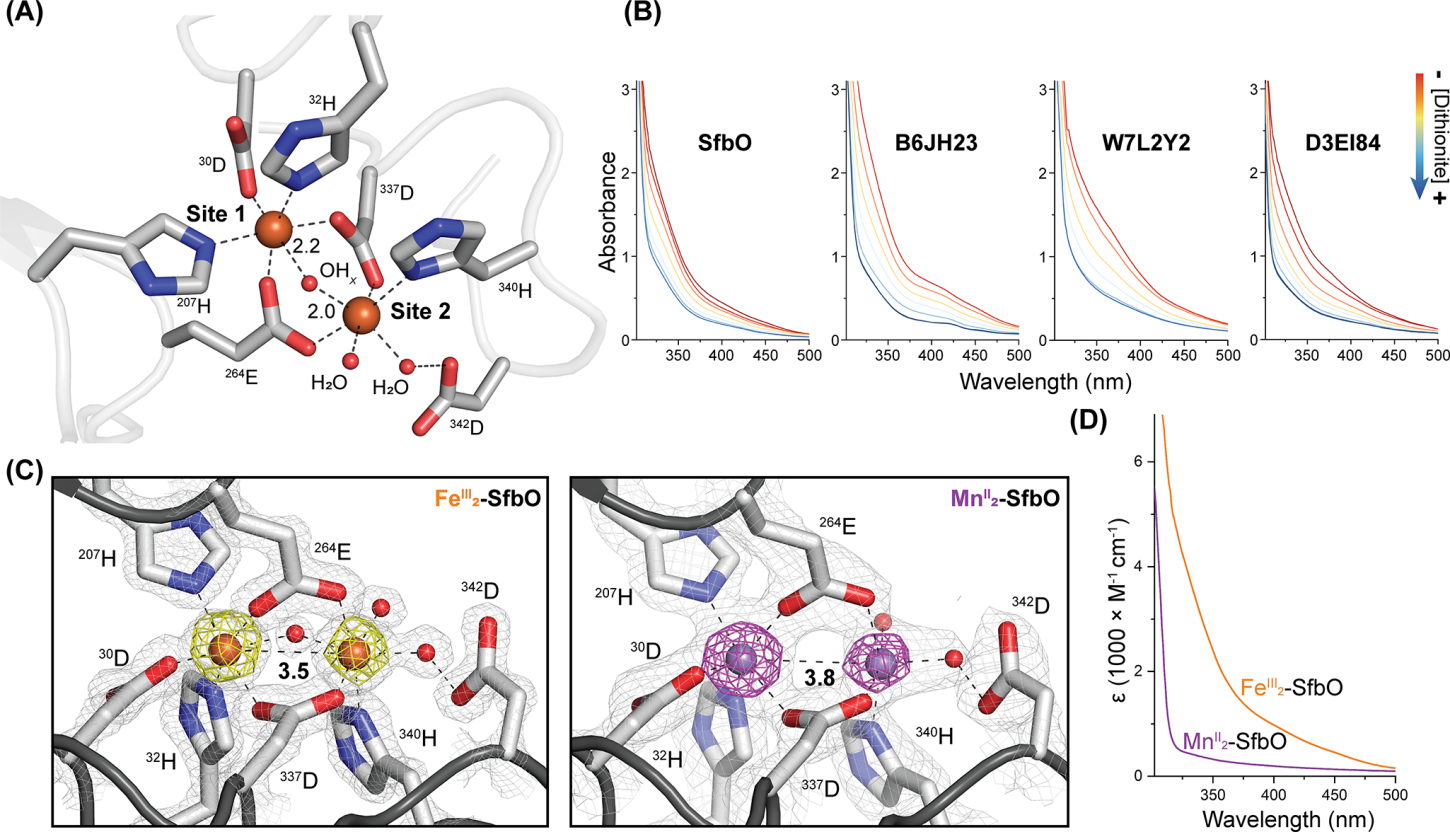
(A) Active site structure of Fe_2_-SfbO. (B) UV/vis traces of Fe^III^_2_-metalated proteins reduced with substoichiometric aliquots of sodium dithionite. (C) Fe^III^_2_- and Mn^II^_2_-metalated SfbO active sites. Gray mesh (2*F*_o_ – *F*_c_: 2.5σ for Fe^III^_2_-SfbO and 1.5σ for Mn^II^_2_-SfbO); yellow mesh (Fe anomalous difference: 18.0σ); purple mesh (Mn anomalous difference: 14.0σ). (D) Comparative UV/vis spectra of the listed SfbO preparations.

Each of the four candidate AROs can harbor redox-active diiron cofactors. Aerobic samples of these Fe_2_-metalated proteins were found to exhibit moderately intense UV/vis features centered near ∼350 nm (Figure 2B), consistent with charge transfer bands classically associated with diferric Fe^III^–OH_*x*_–Fe^III^ motifs.^22^ These features were diminished upon anaerobic addition of ∼1.2 equiv of sodium dithionite (DT) and returned following reexposure of these samples to air (Figure S3). This indicates that the diferrous (Fe^II^_2_) cofactors were readily oxidized by O_2_ and return to a reducible diferric (Fe^III^_2_) resting state rapidly. Indirect structural insight into the cofactor dynamics accompanying the overall redox process was gleaned from a comparison of the active site in its Fe^III^_2_- and Mn^II^_2_-metalated forms of SfbO (Figure 2C). The latter structure was obtained on a single crystal grown under anaerobic conditions and revealed subtle differences from the Fe^III^_2_ congener. The intermetallic distances *d*(M···M) = 3.5 and 3.8 Å found for the Fe^III^_2_ and Mn^II^_2_ forms, respectively, intimate that upon reduction of the Fe^III^_2_ form, the bridging μ-OH_*x*_ ligand is extruded and the metal ions concomitantly adopt five-coordinate square-pyramidal geometries. Consistent with this hypothesis, anaerobic solutions of Mn^II^_2_-metalated SfbO do not exhibit detectable charge transfer bands (Figure 2D).^23^ We thus hypothesize that similar open coordination sites are revealed in the Fe^II^_2_ form of SfbO and that this may facilitate O_2_ coordination and its subsequent multistep reduction to return the metallocofactor to a stable Fe^III^_2_ state.

While the natural substrates of these candidate AROs were not readily inferred from the available genomic data (Figure 1D), we surveyed their ability to perform prototypical monooxygenase reactions in the presence of O_2_, a sacrificial reductant (sodium ascorbate), and a buffering agent. Because of the active site SO_4_^2–^ in the structures of SfbO, we performed a cursory screen of the reactivity of these enzymes toward olefinic and alcoholic substrates containing pendent sulfonate groups (Figure S4) with the intent of observing epoxide and aldehyde products, respectively. In assays performed with vinylsulfonate and isethionate, an ion (*m*/*z* = 123 Da) matching the expected product was observed. However, negative control assays revealed that the buffering agent, 2-(*N*-morpholino)ethanesulfonic acid (MES), was the source of this reaction product (Figure S4B). In principle, the formation of sulfoacetaldehyde (SA) from the MES buffer could stem from the hydroxylation of the *N*-α C–H bond to furnish an unstable hemiaminal product that upon subsequent hydrolysis furnishes SA and morpholine (Figure 3A). Indeed, the SA product was not detectably generated when these assays were performed in the absence of a (2-aminoethylsulfonate)-containing buffer (Figure 3B) or anaerobically (Figure S5), suggesting a requirement for O_2_. Related N- and O-dealkylation reactions are initiated by a variety of cytochrome P450s and Rieske oxygenases which are thought to utilize O_2_-derived high-valent intermediates.^24,25^

**Figure 3:**
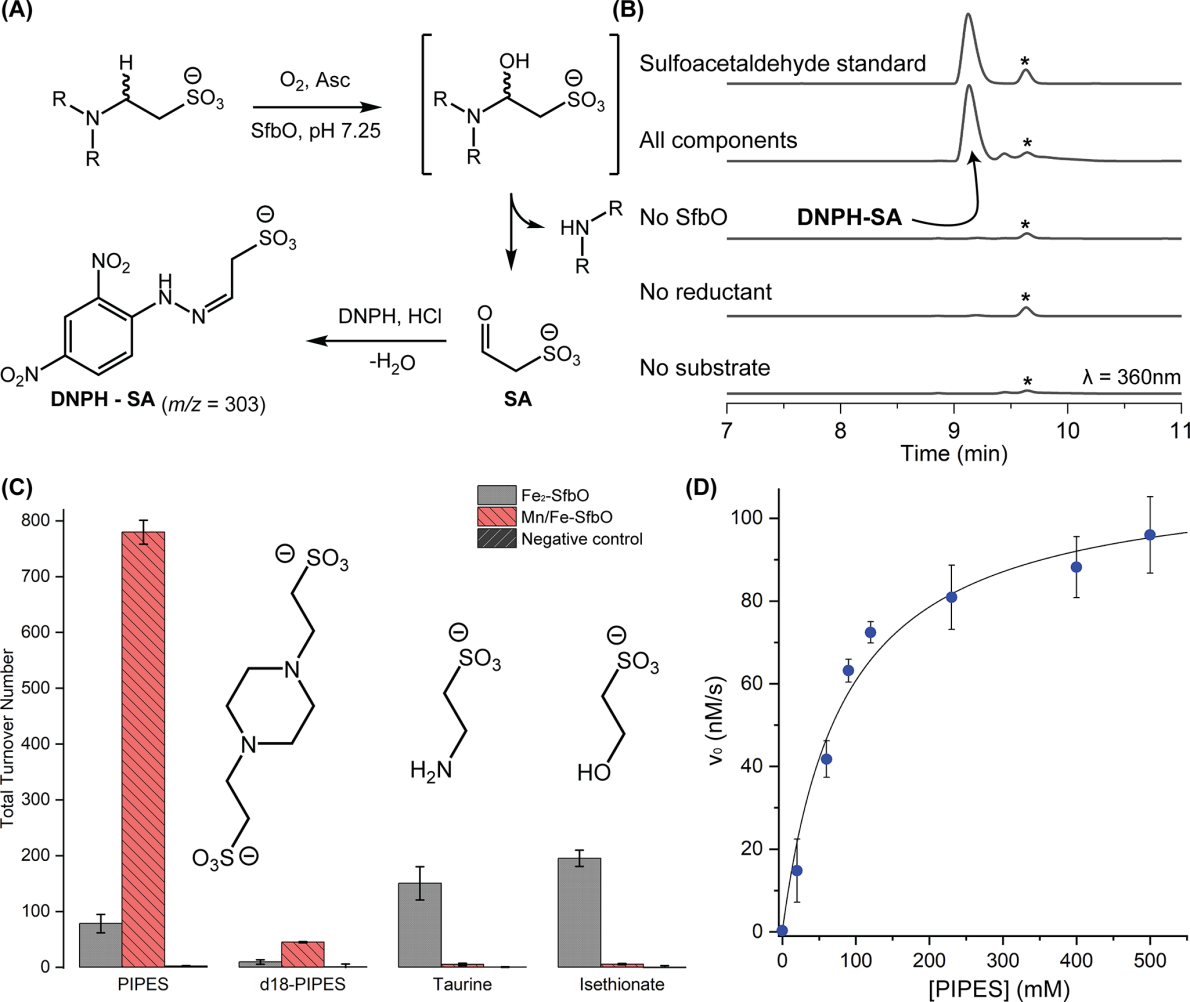
(A) Proposed SfbO-mediated enzymatic reaction and assay workup procedure. (B) Representative UV-HPLC traces of enzymatic assays. * denotes a DNPH-derived impurity. (C) Comparative reactivity of SfbO with listed cofactors and substrates. The negative control shown lacks SfbO. (D) Michaelis–Menten plot of the reaction of Mn/Fe-SfbO with PIPES.

To establish this enzymatic reaction, we then screened the reactivity of SfbO with a handful of 2-aminoethylsulfonate-containing substrates (Figure S5B) and determined that piperazine-*N*,*N*′-bis(2-ethanesulfonic acid) (PIPES) served as the preferred substrate when this enzyme was constituted with a mixed Mn/Fe cofactor. The preparation of the Fe_2_-, Mn_2_-, and Mn/Fe-metalated forms of SfbO is described in the Supporting Information, and their metal contents were ascertained by inductively coupled plasma optical emission spectrometry (ICP-OES) (Table S2). Under identical assay conditions employing the PIPES substrate, Mn/Fe-metalated SfbO yielded >7 times the amount of SA compared with Fe_2_-metalated SfbO, as determined by HPLC analysis (Figure 3C; also see the Supporting Information). Control experiments performed with H_2_O_2_ as an oxidant or in the presence of SOD support O_2_ as the requisite oxidant (Figure S6). The production of SA was substantially diminished when perdeuterated PIPES (*d*_18_-PIPES) was employed as a substrate, suggesting that C–H bond cleavage is (partially) rate-limiting under the assay conditions. In these reactions, *d*_1_-SA was the sole aldehyde-containing product detected, as the expected isotopologue (*d*_3_-SA) undergoes rapid hydrogen/deuterium exchange during assay workup (Figure S5). Interestingly, Mn/Fe-metalated SfbO exhibits a noted preference for the PIPES substrate compared to Fe_2_-metalated SfbO, which was found to convert all of the tested substrates to comparable degrees (Figure 3C). While efficient enzymatic activity was observed for the surrogate substrate PIPES, its binding affinity is low (*K*_M_ = 83 mM and *k*_cat_ = 0.11 s^–1^ for PIPES and Mn/Fe-SfbO; Figure 3D). We thus deem it unlikely that this serendipitous substrate represents the native substrate of SfbO. Nonetheless, the superior hydroxylation efficiency of the Mn/Fe-metalated form of SfbO compared to its Fe_2_ congener for this reaction is notable.

Insight into the electronic and geometric structures of the Mn/Fe cofactor of SfbO was gleaned from electron paramagnetic resonance (EPR) studies. The EPR spectrum of air-oxidized Mn/Fe-SfbO at 15 K contains an intense *S* = 1/2 signal exhibiting hyperfine coupling to a single *I* = 5/2 ^55^Mn nucleus (Figure 4A). Simulations of this signal reveal a quasi-axial **g** tensor (2.026 2.020 2.021) and ^55^ Mn hyperfine coupling tensor **A**_**Mn**_ [(240 360 300) MHz] that collectively implicate the formation of an antiferromagnetically coupled Mn^III^/Fe^III^ cofactor. This hypothesis is bolstered by the spectrum of ^57^Fe-labeled Mn/Fe-SfbO (Figure 4B) which can be simulated with the inclusion of an additional ^57^Fe hyperfine coupling tensor (**A**_**Fe**_ [(−70 −70 −70) MHz]). These EPR signals are comparable to those of related cofactors found in AibH1H2,^9^ class Ic ribonucleotide reductases,^26^ and R2Lox,^27^ which harbor antiferromagnetically coupled Mn/Fe clusters. The addition of either 300 mM sodium sulfate (Figure 4C), or 200 mM PIPES (Figure 4D) to similarly prepared Mn/Fe-SfbO samples resulted in distinct EPR signals. An overlay of these spectra (Figure S7) demonstrates spectral sharpening upon inclusion of these small molecules. Dramatically, upon addition of 20 mM 2-aminoethylphosphonate (AEP), the EPR spectrum of the Mn^III^/Fe^III^ species is grossly perturbed (Figure 4E). These spectral differences manifest simulation parameters (**g** (2.083 2.008 1.964); **A**_**Mn**_ [(240 270 270) MHz]) indicative of increased **g** anisotropy and reduced **A**_**Mn**_ anisotropy. These differences are analogous to those found in the conversion of Mn/Fe R2lox between the resting and I_3_ states and were interpreted as arising from a longer metal–metal distance and a weaker antiferromagnetic coupling between the Mn and Fe centers.^28^ We hence speculate that AEP may coordinate to one or more redox states of the cofactors, whereas the SO_3_^–^-containing molecules occupy sites within the secondary coordination sphere of the cofactor.

**Figure 4:**
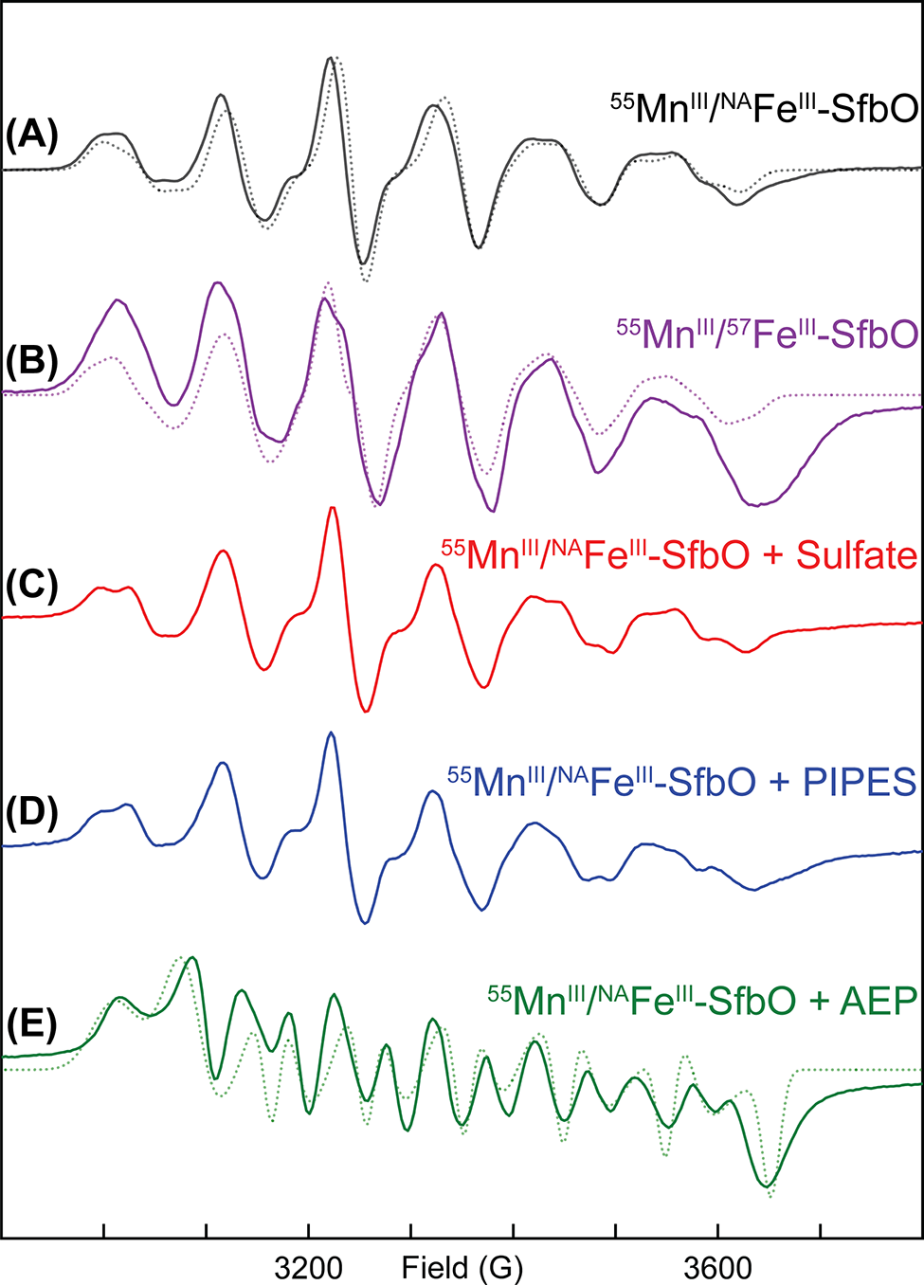
Continuous-wave X-band EPR spectra of (A) Mn/Fe-SfbO, (B) ^57^Fe-labeled Mn/Fe-SfbO, (C) Mn/Fe SfbO in the presence of 300 mM Na_2_SO_4_, (D) Mn/Fe SfbO in the presence of 200 mM PIPES, and (E) Mn/Fe-SfbO in the presence of 20 mM AEP. All spectra were collected at 15 K and 0.6325 mW power.

In summary, we have disclosed a bioinformatic strategy for the identification of new monooxygenases that harnesses bioinorganic logic. One of these proteins, SfbO, was found to mediate a C–H bond hydroxylation reaction in a cofactor-dependent manner. While most studied dinuclear monooxygenases utilize Fe_2_ active sites, the enzymatic activity of Mn/Fe-metalated preparations of SfbO was found to be superior to that of Fe_2_ preparations in terms of both turnover number and selectivity on a substrate surrogate. To the best of our knowledge, a similar cambialistic behavior is unknown for any natural monooxygenase. Hence, the ability of SfbO to function with either an Fe_2_ or Mn/Fe cofactor emphasizes that the unique structural features present in AROs promote unconventional reactivity and warrant further scrutiny.

## Supporting Information Available

Experimental procedures, analytical data, X-ray diffraction data, and EPR simulations and parameters (PDF)
