# Supplementary material for: Bioinformatic Discovery of a Cambialistic Monooxygenase
Source: J Am Chem Soc. 2024 Jan 10;146(3):1783–8. doi: 10.1021/jacs.3c12131 (PMC10811679; doi:10.1021/jacs.3c12131)
Supplement: Supplementary file 1 — ja3c12131_si_001.pdf [file ja3c12131_si_001.pdf]

Supporting Information for

Bioinformatic Discovery of a Cambialistic Monooxygenase

Chang Liu,[1] Magan M. Powell,[1] Guodong Rao,[2] R. David Britt,[2] Jonathan Rittle[1*]

[1]Department of Chemistry, University of California, Berkeley; Berkeley, CA 94720, USA

[2]Department of Chemistry, University of California, Davis; Davis, CA 95616, USA

*Corresponding author. Email: rittle@berkeley.edu

Methods

General. All chemicals were purchased from commercial suppliers and used directly as received unless noted otherwise. Crystallization reagents were purchased from Hampton. All aqueous solutions were prepared using water purified by a MilliQ Academic water purifier and exhibited a conductivity of 18.2 MΩ. Large-scale (>0.5 L) bacterial cultures were grown using a LEX-48 bioreactor (Epiphyte3). All degassed chemicals were rendered anaerobic by equilibration overnight in a Coy anaerobic chamber equipped with a 1.5-2.5% H_2/N_2 environment and a palladium catalyst. Metal analysis was conducted in the Microanalytical Facility in the College of Chemistry at the University of California, Berkeley using a Perkin Elmer ICP Optima 7000 DV Spectrometer. Analytical HPLC was performed on an Agilent 1260 Infinity LC equipped with an Agilent Eclipse Plus C18 column (4.6 * 250 mm, 5 µm). LC-MS analysis was performed on a Waters UHPLC-MS system equipped with an XBridge C18 column (4.6 * 100 mm, 3.5 µm). UV-visible absorption spectra were obtained on an Agilent HP 8453 UV/Vis Spectrophotometer. Nuclear Magnetic Resonance (NMR) spectra were collected on a Bruker NEO-500 spectrometer at the University of California, Berkeley College of Chemistry NMR Facility.

Bioinformatic analyses. Sequence similarity networks (SSNs) were generated with the EFI-EST(1) server, visualized and edited in Cytoscape v3.8.2.(2) Genome neighborhoods were evaluated using the EFI-GNT server(1) and analyzed with Cytoscape v3.8.2, with the statistics visualized with OriginPro (OriginLab Corporation, version 2021). The sequences used for construction of SSNs and GNN analysis were retrieved from UniProtKB for PF00355 (June 2022). The node and edge tables of the GNN were used to determine the list of genes adjacent to small (<250 amino acids) PF00355 proteins (neighborhood size = 3) to achieve the visualization of Fig 1B and the statistics in Fig 1C.

General expression protocol for ARO proteins. The genes encoding the AROs of interest were codon optimized for *E. coli* and ordered as gene blocks from Twist Bioscience, carrying NdeI and XhoI restriction sites for plasmid construction. The gene blocks were inserted into a pET28a vector fused with a N-terminal His_6-tag separated from the ARO coding sequence by a TEV protease cleavage sequence. The plasmid was transformed into *E. coli* BL21 (DE3) cells, and a single colony was used to inoculate 20 mL lysogeny broth (LB) containing 50 µg/ml kanamycin, and shaken at 250 rpm overnight at 37 °C. M9 minimal medium was generally used for large-scale protein growth and contained MilliQ water supplemented with 70 mM phosphate (KH_2PO_4 / Na_2HPO_4), 8.6 mM NaCl, and 18.7 mM NH_4Cl. The pH of these solutions was adjusted with NaOH to pH 7.4 and autoclaved followed by the addition of 900 µM $MgSO_4$, 75 µM $CaCl_2$, and 0.375% glucose from filtered stock solutions. The starter culture was used to inoculate 2 L of M9 medium and grown immersed in a water bath set to 38 °C and vigorously aerated with HEPA-filtered compressed air. At an $OD_{600} \approx 0.6$, the water bath temperature was decreased to 16 °C and 0.125 mM of divalent manganese or iron, in the form of $MnCl_2 \cdot 4H_2O$ or $(NH_4)_2Fe(SO_4)_2(H_2O)_6$, respectively, and 0.5 mM isopropyl β-D-1-thiogalactopyranoside (IPTG) were added immediately. Cultures were grown at 16 °C overnight and then harvested and frozen at -80 °C until purification.

Purification protocol for ARO proteins. All protein purification steps were performed at 4 °C. Thawed cell pellets were resuspended with 10 mL lysis buffer (200 mM NaCl, 50 mM HEPES pH 8.0) per 1 L culture of cells. Cells were lysed by sonication (15 min total pulse time, 3 seconds on, 3 seconds off). The lysate was centrifuged at 12,000 rpm for 40 min at 4 °C to remove cell debris and the resulting supernatant was loaded onto a column equipped with Ni-NTA resin (Thermo Fisher Scientific). The resin was washed with 200 mL lysis buffer, and 700 mL wash buffer (lysis buffer + 25 mM imidazole), and eluted with 200 mL elution buffer (lysis buffer + 250 mM imidazole). The protein was concentrated to ~40 mL using an Amicon® stirred cell (Millipore Sigma) equipped with a 30 kDa filter and subsequently buffer exchanged into 20 mM HEPES pH 7.5 via dialysis. Protein purity was evaluated via sodium dodecyl sulfate–polyacrylamide gel electrophoresis (SDS-PAGE (Fig S7). Protein concentrations were determined from the absorbance at 280 nm calculated based on their molar absorption constant. The calculated molar concentrations are relative to the protein monomers.

For SfbO preparations, the N-terminal His_6-tag was removed with TEV protease by stirring the protein in lysis buffer at a final concentration of 1 mg/mL with 2.5 mM dithiothreitol and 1 mg of TEV protease for every 10 mg of SfbO overnight at 4 °C. The resulting solution was filtered and passed down a Ni-NTA affinity column, and the flow-through collected and buffer exchanged to 20 mM HEPES pH 7.5 via dialysis

as described above. The resultant protein was used for crystallography and reaction assays. The His_6-tag of the other 3 AROs was not removed.

Dithionite titration and oxygen reactivity of Fe-containing ARO proteins. Aerobically-purified protein solutions were rendered anaerobic following repeated evacuation and purging with N_2 and subsequently allowed to equilibrate for at least 1 h in a Coy chamber. These samples were transferred to an air-tight cuvette amenable for UV/visible absorption studies. UV/visible spectra of these solutions revealed characteristic Fe^{III}-OH_x charge transfer bands (Fig 2B). Subsequent spectra were recorded during the addition of substoichiometric aliquots of sodium dithionite and incubated for 5 min before each successive spectrum was recorded. Absorbance values were normalized to account for dilution. Following the titration endpoint, the cuvette was opened to air and gently mixed via hand shaking to aerate the solution. Subsequent UV-visible spectra were taken over 20 minutes to observe the regeneration of the Fe^{III}-OH_x charge transfer bands.

Crystallization of AROs. Single crystals of all proteins were obtained by sitting-drop vapor diffusion at room temperature. Each reservoir was filled with the condition solution (200 µL), and 2 µL of this solution was added to the well containing 2 µL of the protein sample. All proteins (except the Mn_2-metallated form of SfbO) were crystallized in the presence of a two-fold excess of Fe^{II} to ensure a diiron active site constitution (initial protein samples were obtained via expression in M9 minimal medium with 0.125 mM Fe^{II}). All crystals were harvested and frozen in liquid N_2. The specific crystallization conditions employed are listed below

SfbO (8 mg/mL initial protein concentration) was crystallized with a condition solution consisting of 21% PEG 5000 MME, 0.1 M MES pH 6.0, 0.2 M Ammonium sulfate, and 10% v/v glycerol.
Fe_2-SfbO was crystallized aerobically.
Mn^{II}_2-SfbO was crystallized in the presence of a 2-fold molar excess of Mn^{2+} ($MnCl_2 \cdot 4H_2O$) anaerobically using the same crystallization condition.
B6JH23 (11 mg/mL initial protein concentration) was crystallized with a condition solution consisting of 4.2 M 1,6-Hexanediol, 0.1 M BISTRIS pH 6.5, 0.015 M magnesium sulfate, 2% v/v glycerol.
Protein expressed in M9 medium supplemented with additional Fe^{II} was used for crystallization.
W7L2Y2 (9 mg/mL initial protein concentration) was crystallized with a condition solution of 0.07 M Ammonium acetate pH 5.0, 5.6% PEG 4000, and 20% v/v glycerol.
Protein expressed in M9 medium supplemented with additional Fe^{II} was used for crystallization.
D3EI84 (17 mg/mL initial protein concentration) was crystallized with a condition solution of 3.6 M sodium formate, and 10% v/v glycerol.
Protein expressed in M9 medium supplemented with additional Fe^{II} was used for crystallization.

Data processing and structure solution. XRD data were collected at the Advanced Light Source (ALS) and the Stanford Synchrotron Radiation Light source (SSRL). Diffraction data were processed using XDS(3) or iMosfilm(4) followed by scaling and merging using Aimless.(5) Phasing was performed using Phaser-MR via molecular replacement with the homology model generated by SWISS-MODEL(6) based on AibH2 subunit of PDB ID: 6M2I as the initial search model. The models were refined with phenix.refine and manual modeling was performed with COOT. The coordinates, occupancies and b-factors of the metal ions were freely refined. ChimeraX(7) and PyMol were used for visualization. The pdb codes for the described structures are listed below for convenience. SAD experiments were performed on Mn_2- and Fe_2-SfbO at the respective wavelengths (Table S4) to determine the anomalous dispersion response.

SfbO: diiron (8SM6), dimanganese (8SMA)
B6JH23: 8SM8
W7L2Y2: 8SM9
D3EI84: 8SM7

Metalation of SfbO. Homogenously metalated forms of aerobic Fe_2-SfbO preparations (Table S2) were obtained via rapid dialysis of freshly purified samples against 20 mM HEPES pH 7.5 with 10 mM EDTA for 1 h. While the resulting iron content was not significantly diminished by this procedure, the contaminating metals were found to be removed from the as-isolated Fe-SfbO, as judged by ICP-OES.

Access to alternative metalation states of SfbO required the preparation of *apo*-SfbO. Such preparations employed protein expressed in M9 medium supplemented with additional Mn^{II}. The metals found in the resultant, crude Mn-SfbO preparations are entirely removed upon treatment with 5 mM sodium dithionite and 10 mM EDTA for 1 h and subsequent buffer exchange by concentrating the protein sample to ~5 mL using an Amicon® stirred cell and filling with 20 mM HEPES pH 7.5 up to 100 mL five times. This *apo*-SfbO was reconstituted with 2 equivalents of Mn^{II} to furnish Mn-SfbO (Table S2). Overnight incubation of Mn-SfbO with one equivalent of Fe^{II} and subsequent buffer exchange furnished samples of Mn/Fe-SfbO that contain equimolar amounts of each ion (Table S2).

SfbO reaction assay protocol. Enzymatic assays were performed at pH 7.25, and all substrate stocks (1 M or 500 mM) were adjusted to pH = 7.25 with hydrochloric acid or sodium hydroxide. MES, PIPES, HEPES, and TES have good buffering capacity at pH 7.25, so no additional buffer was needed for the reaction assays. Substrate stocks of taurine, isethionate, and CHES were prepared with 5 mM MOPS added for pH adjustment, and those reactions were carried out in 10 mM MOPS pH 7.25. MOPS is not detectably converted by SfbO to generate either sulfoacetaldehye (SA) or sulfopropionaldehyde. Manganese(II) chloride tetrahydrate and ammonium iron(II) sulfate hexahydrate were used to prepare stocks of the supplementary Mn^{II} and Fe^{II} metal ions, respectively.

Standard enzymatic assays (50 μL total volume) contained the listed SfbO preparation (0.5 μM), sodium ascorbate (2 mM), supplementary Fe^{II} or Mn^{II} metal ions (0.25 μM for Fig 3, 1 μM for Fig S5A and B), sulfonate substrate (200 mM), and 0.05 mg/ml catalase where stated. The protein-free controls replaced SfbO with 1 μM each of free Fe^{II} and Mn^{II}. The reagents were combined aerobically and incubated with shaking at 300 rpm and 20 °C for 3 h. Assays were quenched by the addition of a 5 mM DNPH solution in 2 M hydrochloric acid (12 μl), following existing protocols (11). To ensure complete derivatization of the sulfoacetaldehyde product, these mixtures were shaken at 300 rpm at 30 °C for 30 min. The mixture was then neutralized by addition of 2 M NaOH (12 μl), and centrifugated at 16 000 g for 10 min. The yellow solution was then analyzed by analytical HPLC or LCMS. To ascertain the Michaelis-Menton parameters for PIPES hydroxylation, 1 μM of SfbO was employed and reactions were quenched after 25 minutes while under steady-state conditions (Fig S5G).

Synthesis of Sulfoacetaldehyde (SA) Standard. Sodium isethionate (0.125 g, 0.844 mmol) was dissolved in DMSO (1 mL) and combined with 1.5 equivalents of Dess–Martin periodinane (0.537 g, 1.27 mmol) and stirred at 37 °C for 2 h. The resulting mixture was heated to 50 °C and evaporated to dryness *in vacuo* on a Schlenk line. The resultant oily residue was washed with DCM, and the remaining solid was redissolved in water and centrifuged. The clear, colorless supernatant was decanted and dried *in vacuo* overnight at room temperature. The resultant crude sulfoacetaldehyde product was redissolved in water as a stock solution and stored at -80 °C. NMR spectroscopy indicates that the sulfoacetaldehyde is composed of a mixture of the aldehyde and the hydrated geminal-diol. Aldehyde: ^{1}H NMR (400 MHz, D_2O): δ 9.65 (t, *J* = 2.9 Hz, 1H), 4.04 (d, *J* = 2.9 Hz, 2H). Hydrate: ^{1}H NMR (400 MHz, D_2O): δ 5.38 (t, *J* = 5.2 Hz, 1H), 3.17 (d, *J* = 5.2 Hz, 2H). ^{13}C NMR (126 MHz, D_2O): δ 87.85, 58.08. These parameters closely match those found previously.(8) Typical crude yields of 53% do not adequately account for extensive hydration of the product. Analytical NMR quantification suggested that spectroscopic yields are typically in the range of 15%. Sulfoacetaldehyde concentrations were quantified by ^{1}H NMR by addition of a known concentration of phenol standard and calculated based on the integral ratio of the aldehyde- (δ 9.65) and geminal-diol hydrogen (δ 5.38) resonances in sulfoacetaldehyde and the aromatic hydrogen resonances of phenol (Fig S10) (Integrals of the α-Hs (δ 4.04, 3.17) are not suitable for quantification due to their acidity and H/D exchange). The pre-scan delay (D1) was set to 50 s, ensuring a quantitative relationship between the ^{1}H NMR integrals. Mass spectroscopy indicates that two major ions matching with the aldehyde (m/z = 122.95, exact mass = 122.98) and the hydrate (m/z = 140.97, exact mass = 140.99). The sulfoacetaldehyde quantification resulting from ^{1}H NMR is corroborated by the ESI mass spectrum of a mixture of 5.0 mM sulfoacetaldehyde (aldehyde + hydrate) and 5.0 mM sodium allylsulfonate (98 $\pm$ 1% consistent with ^{1}H NMR quantification) (Fig S12). The sulfoacetaldehyde standard was derivatized with 2,4-dinitrophenylhydrazine (DNPH) using the same protocol as the reaction assays and used for calibration curve construction.

HPLC and LC-MS analysis. Liquid chromatography for analytical HPLC was performed using a mixture of water and acetonitrile (MeCN) spiked with 0.1% trifluoroacetic acid. The chromatographic sequence involved a 1 min solvent gradient from 0 – 20% MeCN, a 10 min gradient from 20 – 40% MeCN, a final 1

min gradient from 40 – 100% MeCN and a holding phase at 100% MeCN for 5 min at a constant flow rate of 1 mL min^{-1}. Liquid chromatography for LC-MS was performed using a 10 min solvent gradient from 0 – 100% MeCN and holding at 100% MeCN for 5 min at a flow rate of 1 ml min^{-1}. The usage of this LC-MS data was limited to the detection of DNPH-SA ions (m/z = 303 for DNPH-SA, m/z = 304 for DNPH-d_1-SA), and all product quantifications were based on the integrated absorbance at 360 nm from the HPLC analysis.

Electron Paramagnetic Resonance Spectroscopy. EPR studies were performed in the CalEPR center in the University of California, Davis. X-band continuous-wave EPR spectra were collected on a Bruker Biospin EleXsys E500 spectrometer equipped with a super high Q resonator (ER4122SHQE) in the perpendicular mode. Cryogenic temperatures were controlled by an ESR900 liquid helium cryostat with a temperature controller (Oxford Instrument ITC503). Spectrometer settings were: conversion time, 60 ms; modulation amplitude, 0.8 mT; modulation frequency, 100 kHz. All data was collected at 15 K and 0.6325 mW power. Small amounts of adventitious Mn^{II} signal were subtracted using the corresponding spectrum obtained on an aqueous solution of manganese(II) chloride with the same experimental parameters. EPR spectra were simulated in Matlab with Easyspin 5.2.28 toolbox.(9) The Mn/^{57}Fe-SfbO was reconstituted with the same protocol as Mn/^{NA}Fe except that $^{57}FeCl_2$ was used in place of natural abundance Fe(II) sources.

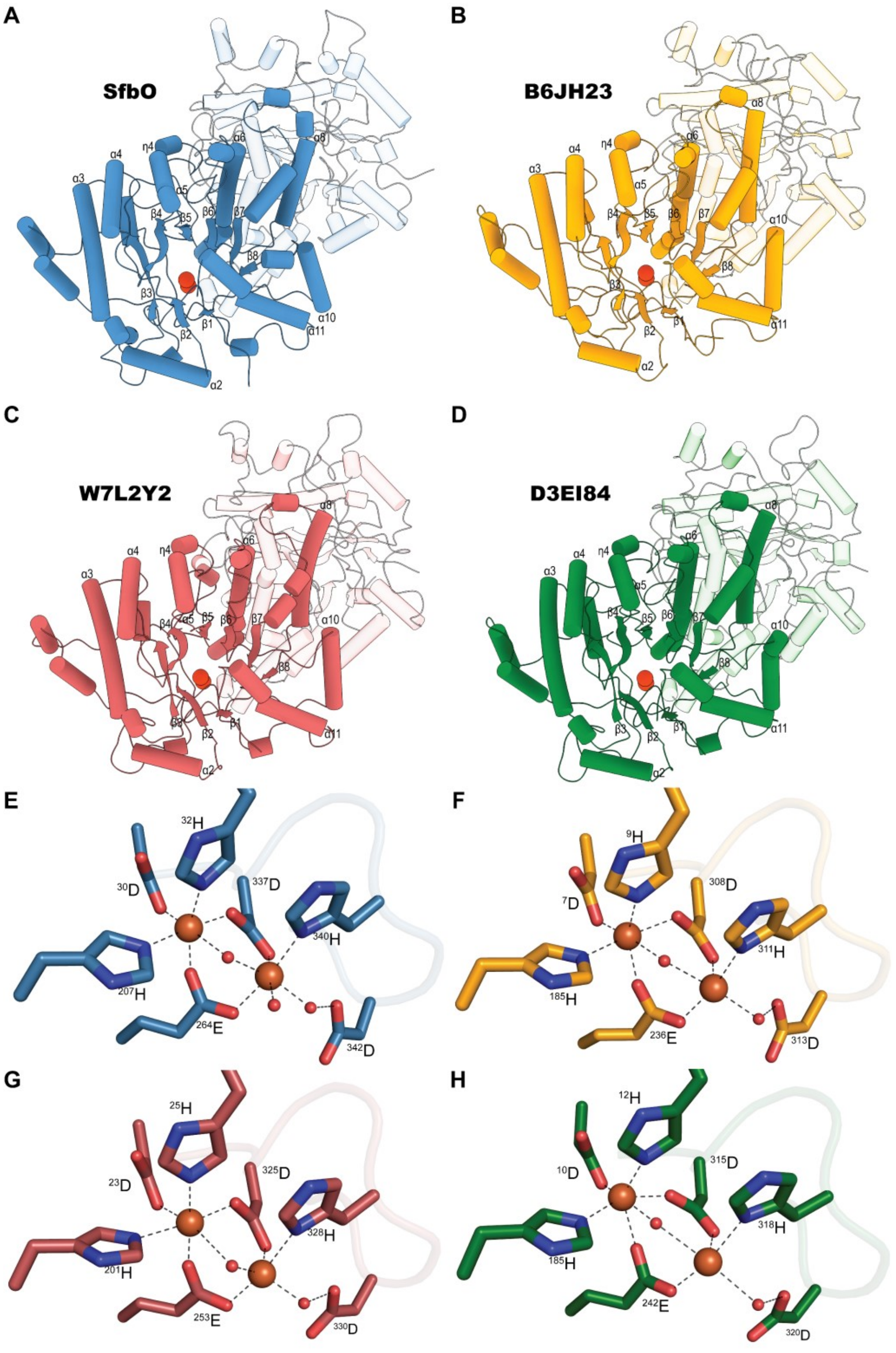

Figure S1. (A-D) Macromolecular structures of selected AROs. Cartoon representations of the structures of SfbO (A), B6JH23 (B), W7L2Y2 (C), and D3EI84 (D). One monomer of each of the homodimeric protein complexes is colored in front. The helices and beta sheets corresponding to the canonical TIM barrel fold are explicitly numbered for clarity. Metal atoms are rendered as orange red spheres. The diiron-metalated active site structures of SfbO (E), B6JH23 (F), W7L2Y2 (G), D3EI84 (H).

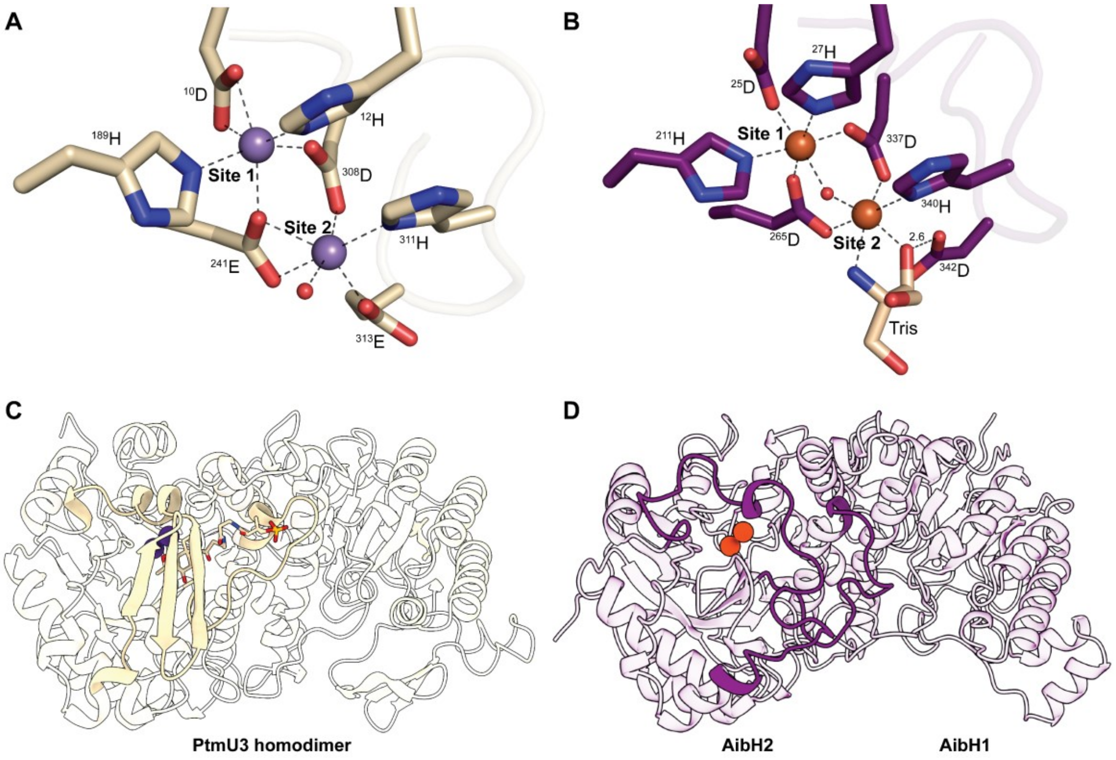

Figure S2. Structures of PtmU3 and AibH1H2. (A) Dimanganese form of PtmU3 (6OMP). (B) Diiron form of AibH1H2 (8FUM). A molecule of Tris coordinates the Site 2 metal via its amino and hydroxyl groups. (C) Amino acid residues that constitute a substrate binding pocket (highlighted in beige) in PtmU3 bound with PTM substrate (PDB 6OMQ). (D) Amino acid residues that constitute a substrate binding pocket (highlighted in dark purple) of AibH1H2 (PDB 8FUM).

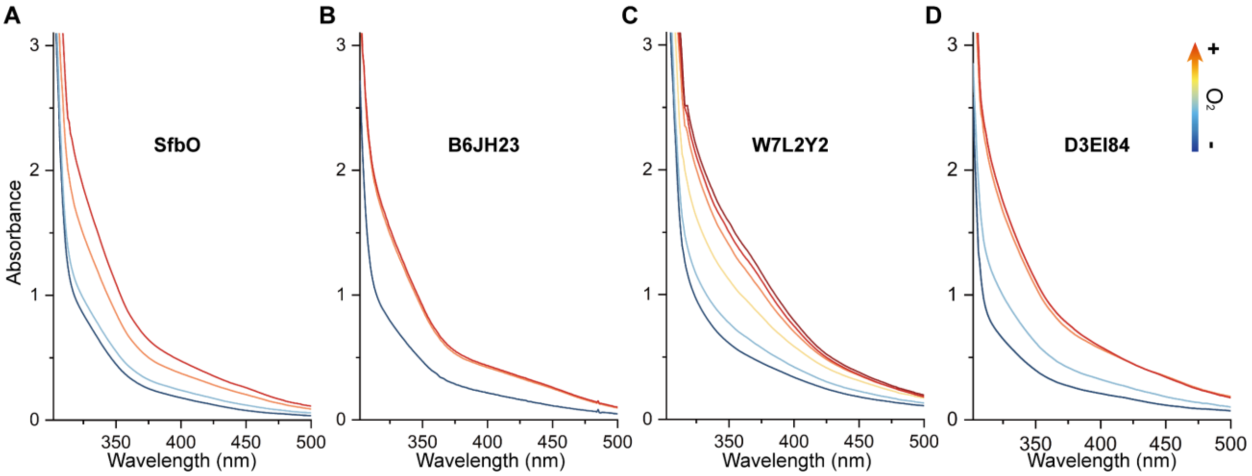

Figure S3. UV/visible traces demonstrating the formation of the Fe^{III}-OH_x-Fe^{III} LMCT bands (complementary to Fig 2B) upon exposure to air. Spectra were taken at intervals of 30 seconds until saturation.

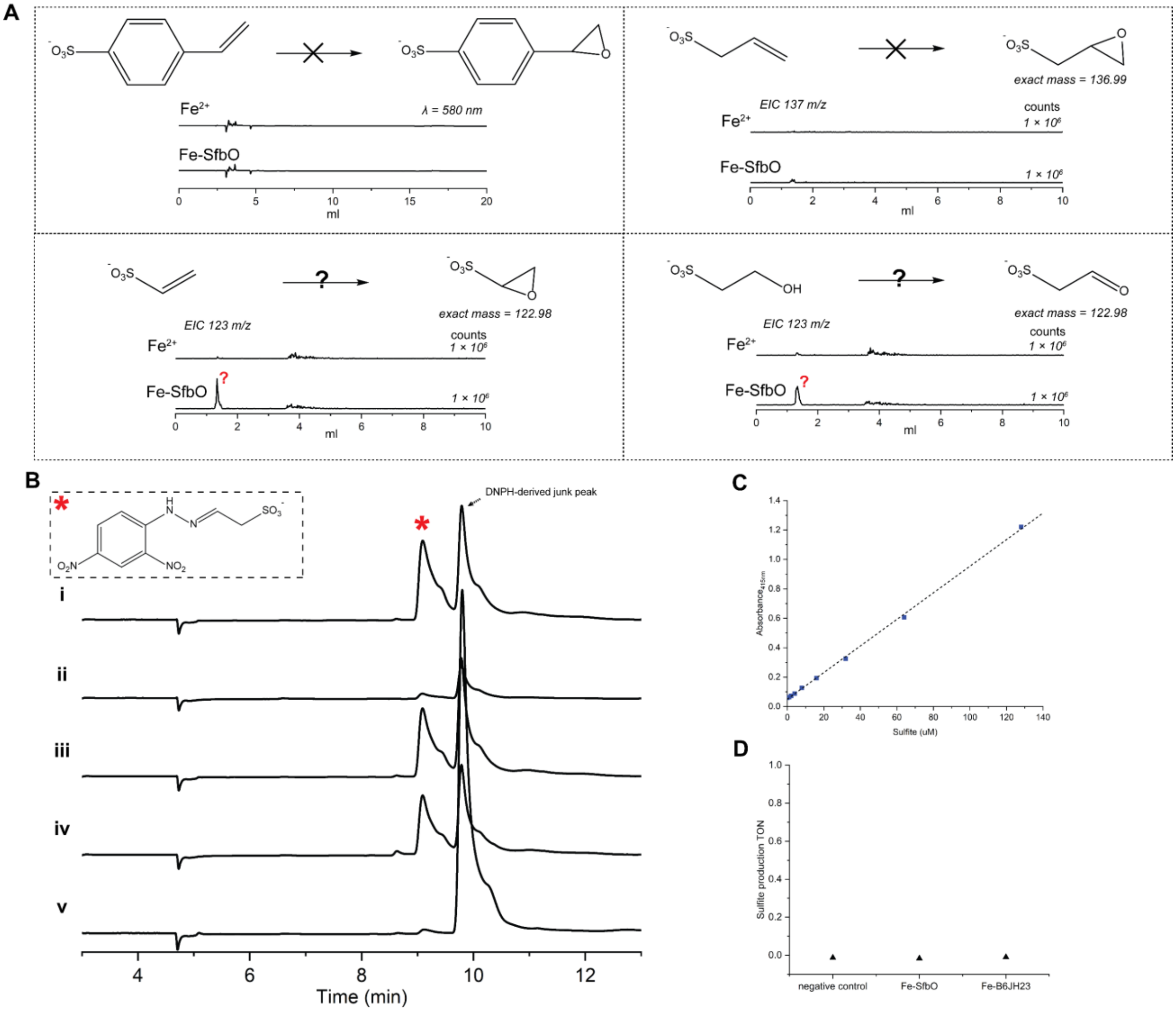

Figure S4. Discovery of SfbO reactivity on MES. (A) Reactivity trials for Fe-SfbO on small molecules containing alkene group at different positions, and isethionate. The trial assays of styrene sulfonate were derivatized via a colorimetric NBP assay for epoxide detection (12) and analyzed with HPLC. The trial assays of allylsulfonate, vinylsulfonate and isethionate were analyzed by LCMS. A product eluting at 1.7 mL exhibited an m/z = 123 was observed for enzymatic reactions of vinylsulfonate and isethionate. (B) Single turnover assays were performed on Fe-SfbO and analyzed with HPLC. The air-oxidized protein was reduced with 10 mM dithionite anaerobically, and the excessive dithionite was removed by buffer exchange. The assay mixtures were prepared anaerobically first and exposed to air for 20 min before quenching by the DNPH derivatization method. (i) A reaction containing 200 μM reduced Fe-SfbO, 200 μM supplementary Fe^{II}, 400 μM sodium isethionate, and 50 mM MES at pH = 6.5. (ii) Assay mixtures with the Fe-SfbO excluded. (iii) Assay mixtures with the supplementary Fe^{II} excluded. (iv) Assay mixtures with the sodium isethionate excluded. (v) Assay mixtures with the MES excluded. The asterisk peak (*) at retention time of 9.1 min is the DNPH-SA product. The peak at 10 min comes from a consistently-observed, unknown DNPH-derived impurity. (C) and (D) The potential alpha C-H bond hydroxylation product is sulfite, which was not observed in the reaction of Fe-SfbO or Fe-B6JH23 with MES or taurine. (C) The calibration curve used for sulfite quantification by using Ellman's reagent. (10) (D) Reactions containing 50 μM enzymes, 5 mM taurine, 50 mM MES pH = 6.5, 100 uM supplementary Fe^{II}, and 5 mM sodium ascorbate. The enzyme in the negative control assay is replaced by 100 uM Fe^{II}. The assays were allowed to incubate at room temperature for 3 h before being analyzed by the Ellman's reagent.

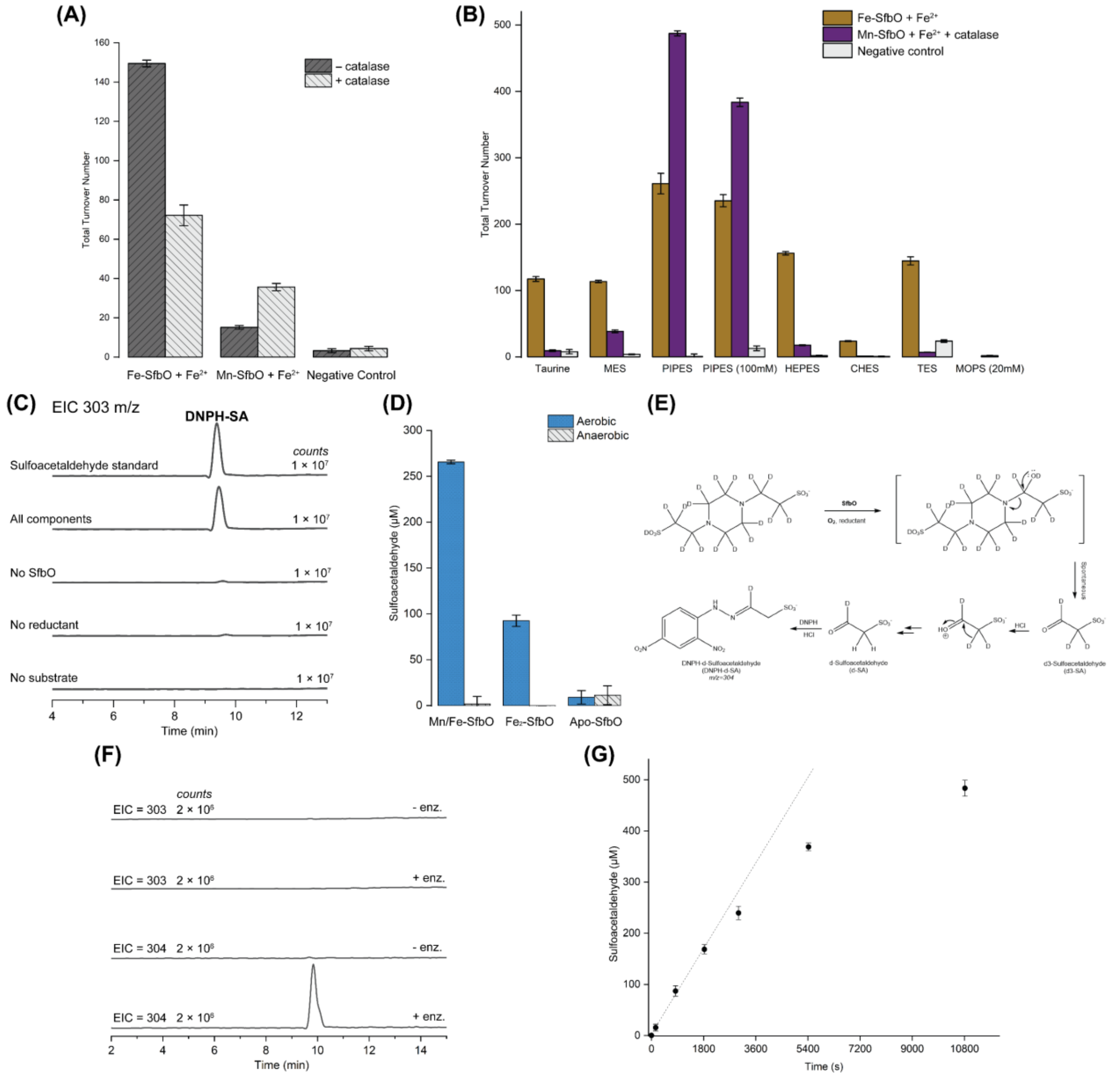

Figure S5. (A) Reactivity of as-isolated Fe-SfbO and Mn-SfbO (0.5 μM) supplemented with additional Fe^{II} (1 μM) towards the oxidative conversion of MES to 2-sulfoacetaldehyde. The presence of catalase was found to markedly influence the reaction outcome. Reactions were performed in triplicate. (B) Substrate scope tests of the reactivity of as-isolated Fe-SfbO and Mn-SfbO with additional Fe^{II} with the listed sulfonate-containing substrates (200 mM if not labeled specifically). Negative controls lack SfbO and contain 1 μM free Fe^{II} and Mn^{II}. SA was not detected from the reaction of SfbO with 20 mM MOPS that was used as buffer. (C) Representative LC-MS traces of enzymatic assays. The DNPH-SA product ion (m/z = 303) was observed only when all reaction components are included. (D) Assays of Mn/Fe-SfbO (0.5 μM), Fe_2-SfbO (1 μM), and Apo-SfbO (1 μM) with 200 mM PIPES incubated without shaking at room temperature anaerobically or aerobically. All reaction components were degassed for at least one hour. No significant product formation was detected in the anaerobic reactions or the reactions of Apo-SfbO. (E) Schematic rationalizing the formation of d^1-sulfoacetaldehyde (d^1-SA) in assays of Mn/Fe-SfbO with d^{18}-PIPES. The initially-formed d^3-sulfoacetaldehyde product is rapidly converted to d^1-SA via hydrogen-deuterium exchange under the acidic workup conditions required for DNPA derivatization. (F) DNPH-functionalized d^1-SA (m/z = 304) is the sole product detected in the reaction of Mn/Fe-SfbO with d^{18}-PIPES. No DNPH-SA (m/z = 303) was detected, confirming that SA generation is derived from the PIPES substrate. (G) The formation rate of SA by Mn/Fe-SfbO is linear for the first 30 minutes of the assay (R^2 = 0.996).

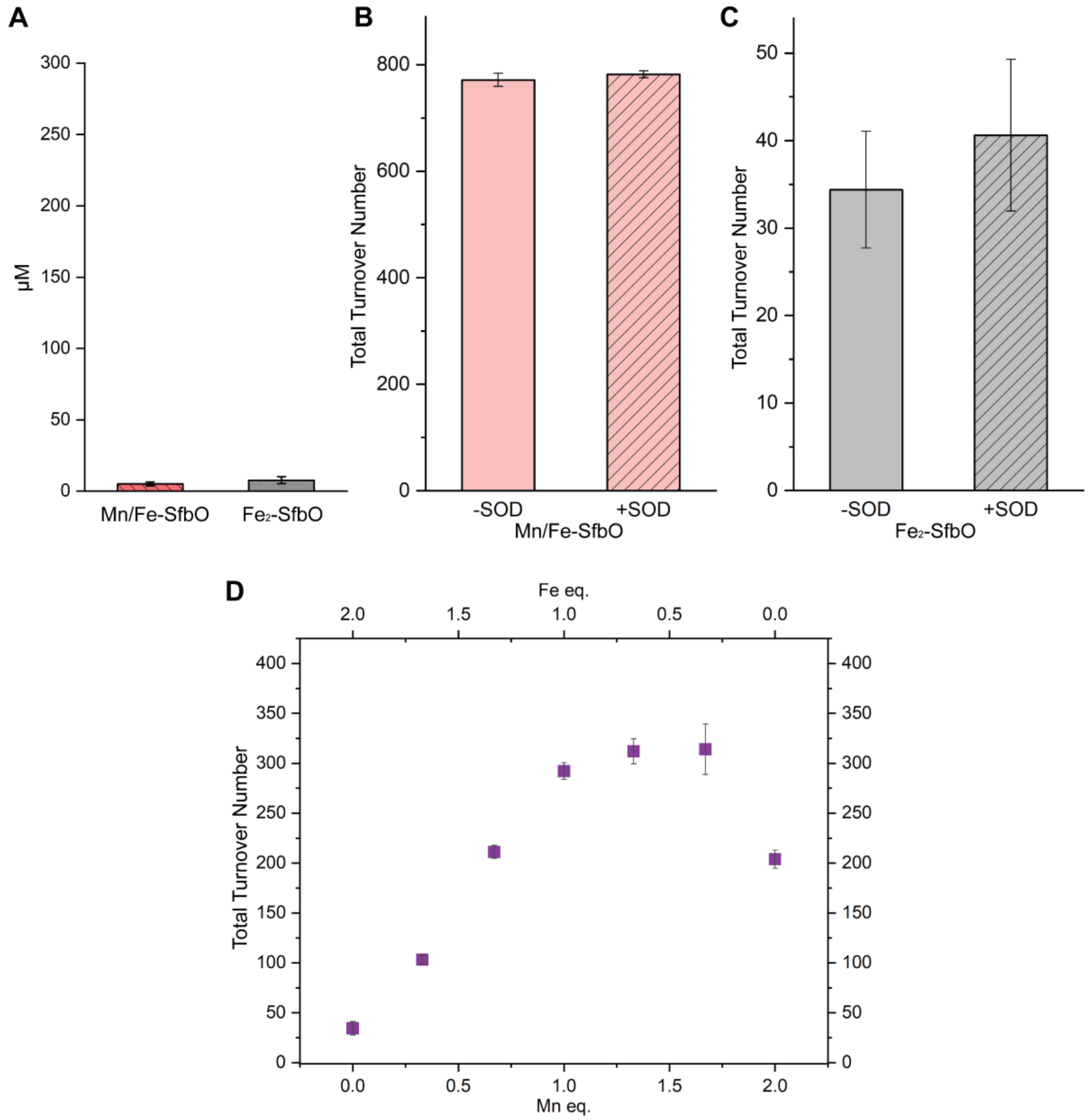

Figure S6. (A) Anaerobic reaction assays of 1 μM Mn/Fe- and Fe_2- SfbO with 200 mM PIPES in the presence of 2 mM H_2O_2. These assays were set up in an anaerobic chamber and incubated at room temperature for 3 h. No significant production SA was detected. (B) Reaction assays of Mn/Fe-SfbO and (C) Fe_2-SfbO with 200 mM PIPES in the presence of catalase were performed with or without addition of 0.1 mg/ml bovine Cu/Zn superoxide dismutase (SOD). No significant activity alteration was observed with the addition of SOD. (D) Dependence of the catalytic activity of SfbO on Mn^{II} and Fe^{II} at a total metal equivalency of 2 per monomer. Premixed metal stocks containing different ratios of Mn^{2+} and Fe^{2+} were added to Apo-SfbO and incubated at room temperature anaerobically for 1 h before the reaction assays were performed. The results indicate that the heterometallic Mn/Fe form is the most active cofactor for this substrate.

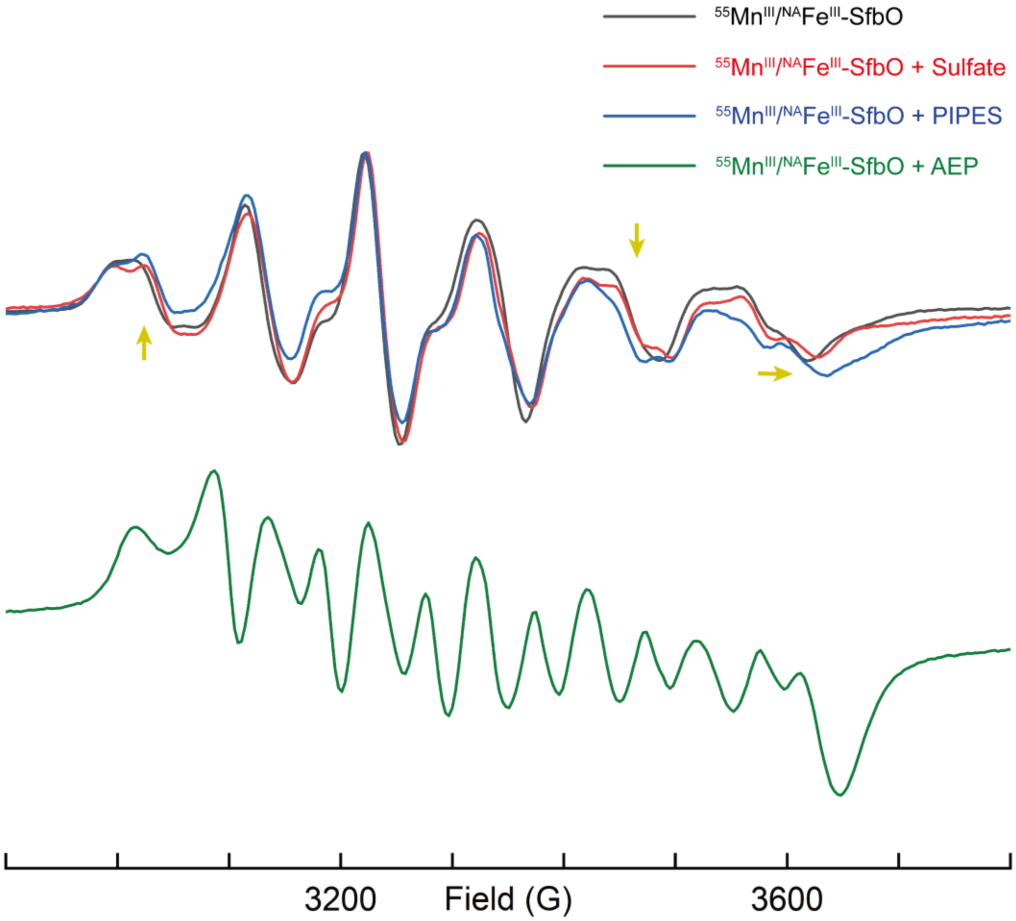

Figure S7. Overlay of the EPR spectra of Mn/Fe-SfbO collected in the presence of 300 mM sodium sulfate, 200 mM PIPES, or 20 mM 2-aminoethylphosphonate (AEP)

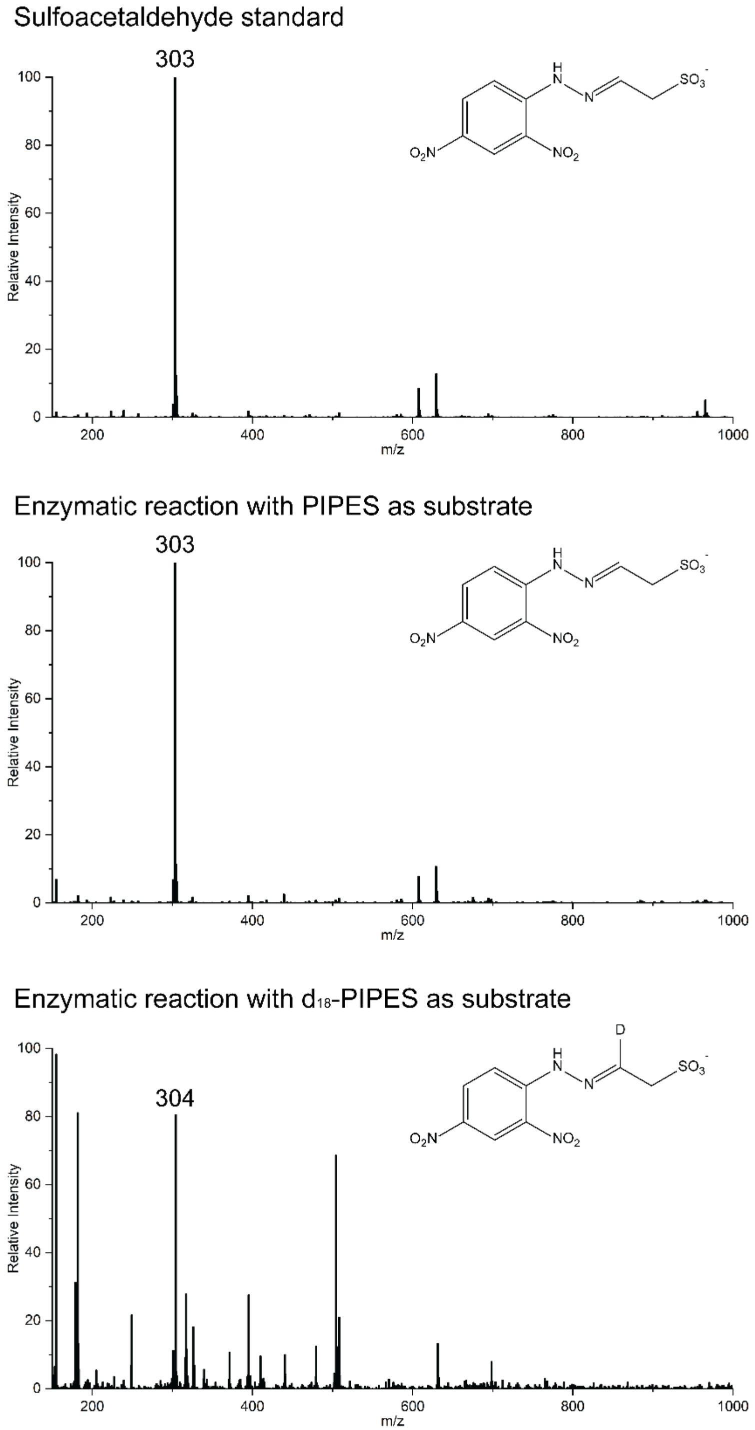

Figure S8. Mass spectra obtained at retention times of 9.7 min in the LC-MS analysis for the sulfoacetaldehyde standard (top), the Mn/Fe-SfbO reaction with PIPES (middle), and the Mn/Fe-SfbO reaction with d_{18}-PIPES (bottom) derivatized with DNPH.

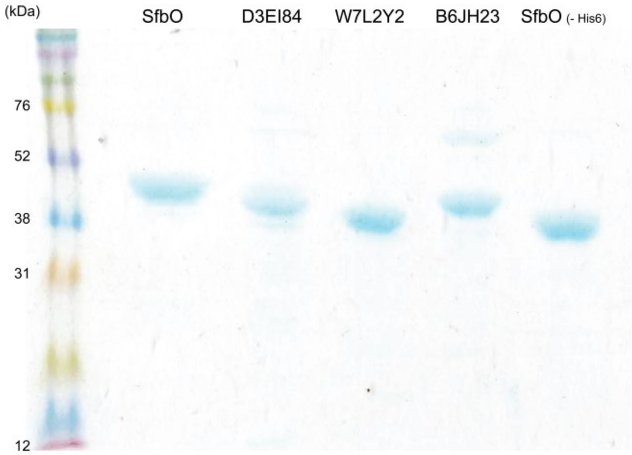

Figure S9. Representative SDS-PAGE gel of SfbO, D3EI84, W7L2Y2, B6JH23 containing His6 affinity tag, plus SfbO with the His6 tag removed by TEV protease processing (left to right).

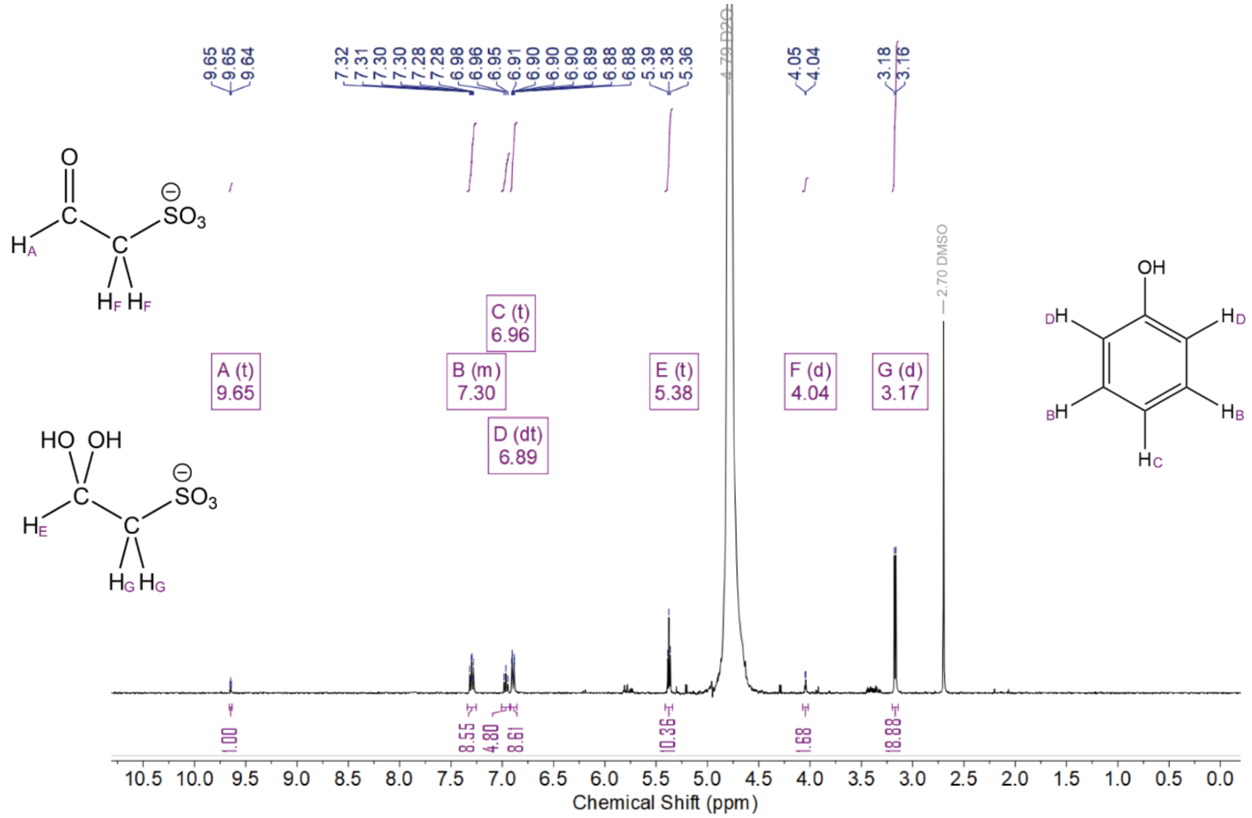

Figure S10. ^{1}H NMR spectrum of sulfoacetaldehyde mixed with 3.33 mM phenol in D_2O (rt, 400 MHz).

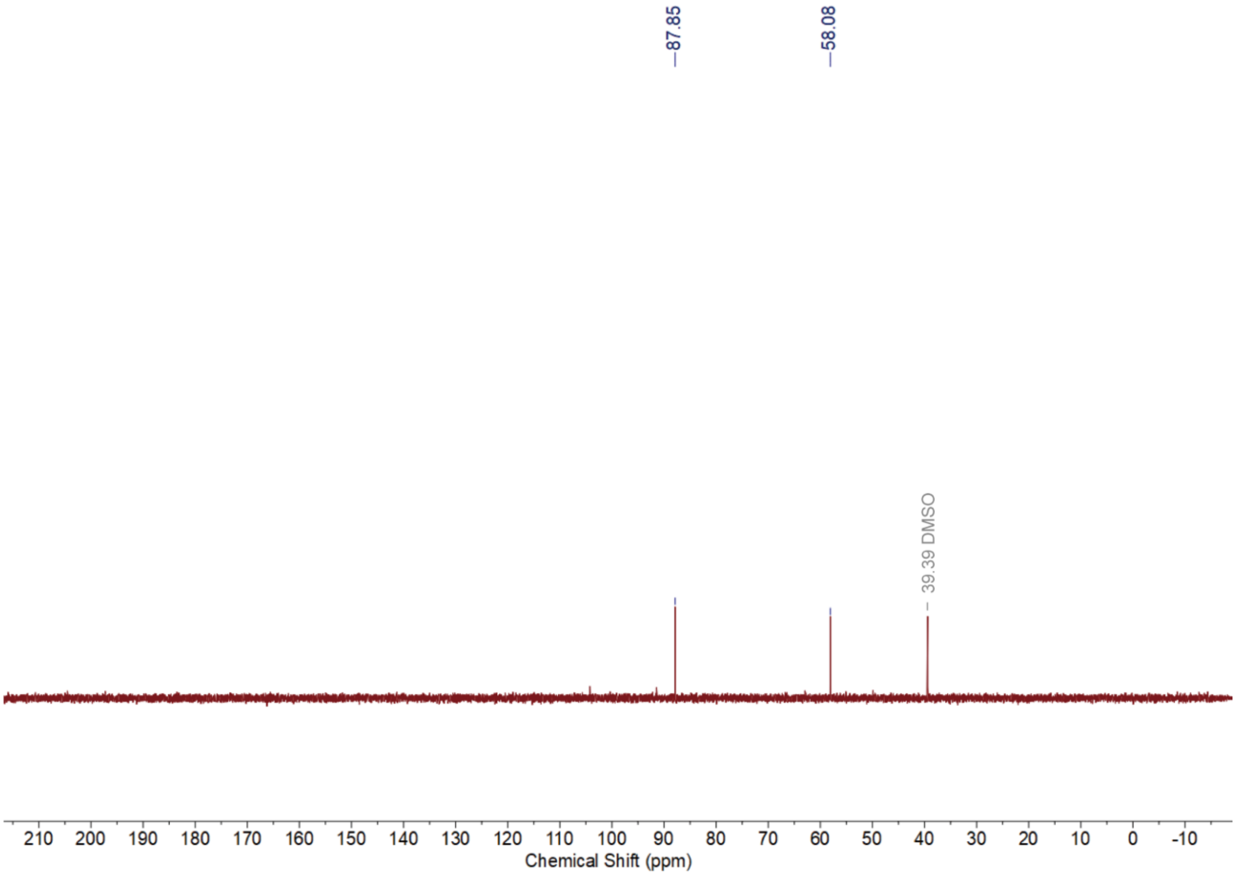

Figure S11. ^{13}C NMR spectrum of sulfoacetaldehyde in D_2O (rt, 126 MHz). Note: the observed resonances correspond to the geminal-diol form of sulfoacetaldehyde.

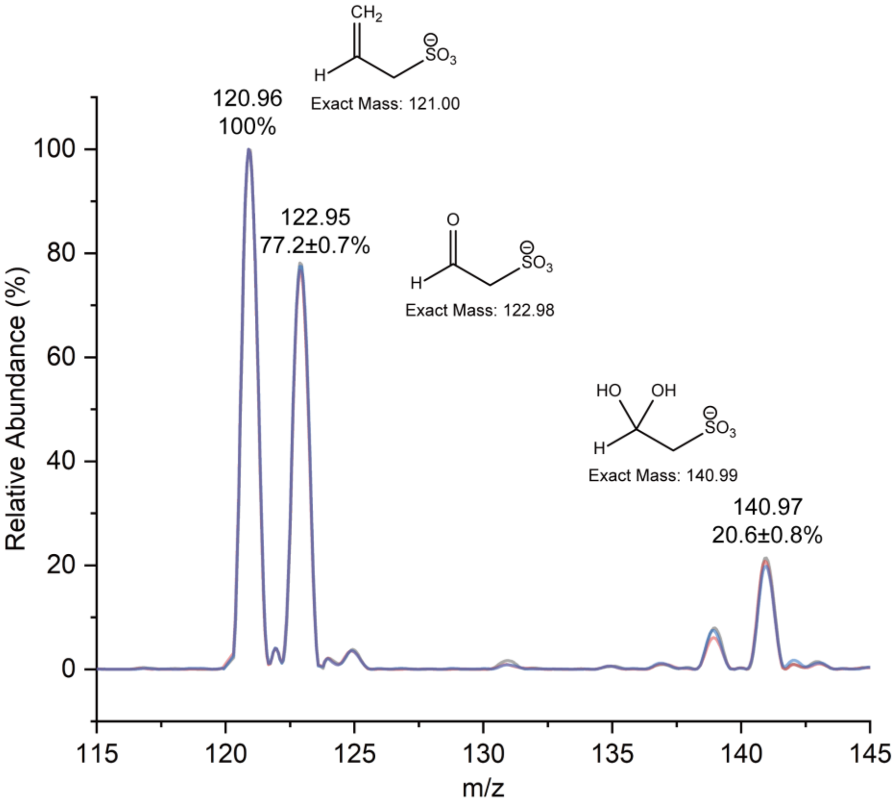

Figure S12. ESI Mass spectrum resulting from a neutral solution containing 5.0 mM sulfoacetaldehyde (aldehyde + hydrate) and 5.0 mM sodium allylsulfonate. The relative abundance of allylsulfonate ion (m/z = 120.96) is set to 100% for comparison with sulfoacetaldehyde ions. Experiment was performed in triplicate (black, red, blue traces).

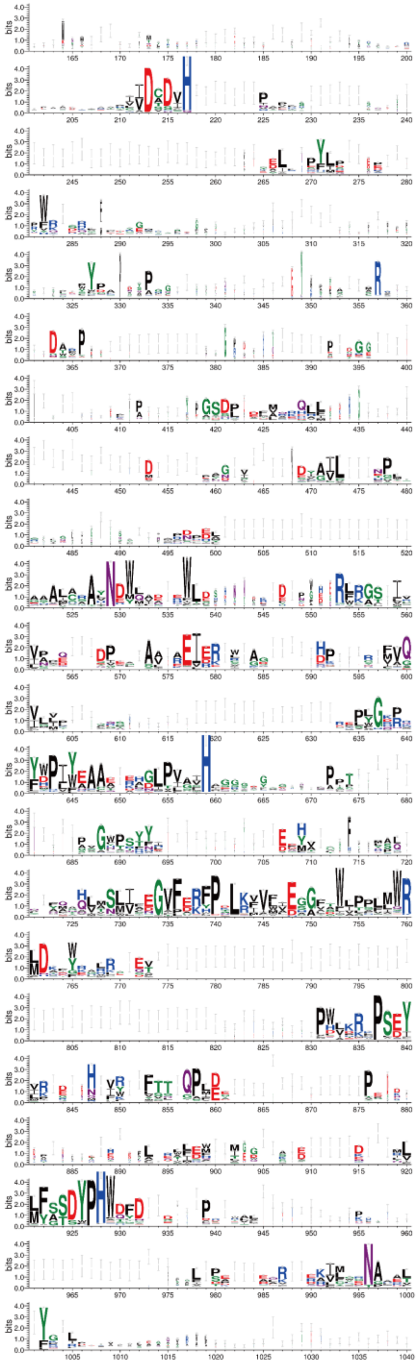

Figure S13. Multiple sequence alignment of candidate AROs (Table S5). Weblogo 3 was used for visualization (13). The range of sequence identity is 25 - 100%.

Rank	Pfam	Pfam description	Function of representative enzymes	Annotation	Number of genes
1	PF00032-PF00033	Cytochrome b(C-terminal)/b6/petD-Cytochrome b/b6/petB	Oxidoreductase	**Oxidoreductase**	**11478**
2	PF02167	Cytochrome C1 family	Oxidoreductase	**Oxidoreductase**	**9737**
3	PF00005	ABC transporter	Substrate transporter	**Other**	**5035**
4	PF01458-PF19295	SUF system FeS cluster assembly, SufBD-SufBD protein N-terminal region	FeS cluster assembly	**FeS cluster assembly**	**4477**
5	PF00266	Aminotransferase class-V	Cysteine desulfurase, FeS assembly	**FeS cluster assembly**	**3920**
6	PF01458	SUF system FeS cluster assembly, SufBD	FeS cluster assembly	**FeS cluster assembly**	**3737**
7	PF07992-PF14759	Pyridine nucleotide-disulphide oxidoreductase-Reductase C-terminal	Oxidoreductase	**Oxidoreductase**	**3683**
8	PF01592	NifU-like N terminal domain	FeS cluster assembly	**FeS cluster assembly**	**3488**
9	PF04909	Amidohydrolase	Decarboxylase, Hydroxylase	**Amidohydrolase**	**3307**
10	PF00043-PF13417	Glutathione S-transferase, C-terminal domain-Glutathione S-transferase, N-terminal domain	Glutathione S-transferase	**Other**	**3202**
11	PF00033	Cytochrome b/b6/petB	Oxidoreductase	**Oxidoreductase**	**3115**
12	PF00355-PF00848	Rieske [2Fe-2S] domain-Ring hydroxylating alpha subunit (catalytic domain)	Oxidoreductase	**Oxidoreductase**	**2791**
13	PF00380	Ribosomal protein S9/S16	Ribosome	**Other**	**2608**
14	PF00572	Ribosomal protein L13	Ribosome	**Other**	**2556**
15	PF00126-PF03466	Bacterial regulatory helix-turn-helix protein, lysR family-LysR substrate binding domain	Transcription regulator	**Other**	**1978**

Table S1. Details and coloring scheme of the most conserved genes within three coding regions of the small Rieske proteins present in Fig 1B.

	Metal per monomer			
	Fe	Mn	Ni	Zn
${}^{as\text{-}iso}$Fe-SfbO	1.74 ± 0.03	0.073 ± 0.001	0.118 ± 0.002	0
Fe_2-SfbO	1.61 ± 0.03	0	0	0
Apo-SfbO	0	0	0	0
Mn-SfbO	0	1.15 ± 0.01	0	0
Mn/Fe-SfbO	0.93 ± 0.02	0.84 ± 0.03	0	0

Table S2. Observed metal content of differentially-metalated SfbO samples. Values smaller than the detection limit (0.01 ppm) of the ICP-OES instrument are reported as zero. Samples of ${}^{as\text{-}iso}$Fe-SfbO were obtained following crude protein purification were not subject to any chelation. Each sample was measured by ICP-OES at least twice.

**Pairwise Identity %**	SfbO	D3EI84	B6JH23	W7L2Y2
SfbO	100.0			
D3EI84	40.0	100.0		
B6JH23	34.2	36.7	100.0	
W7L2Y2	35.8	37.4	43.6	100.0

Table S3. Pairwise sequence identity between the 4 featured AROs.

	Fe-SfbO	Fe-SfbO	Mn-SfbO
PDB ID	8SM6		8SMA
Wavelength (Å)	0.979	1.738	1.893
Resolution range (Å)	48.32 - 1.39 (1.44 - 1.39)	73.09 - 2.455 (2.543 - 2.455)	93.53 - 2.18 (2.258 - 2.18)
Space group	P 21 21 21	P 21 21 21	P 21 21 21
Unit cell	[illegible]6.333 128.501 138.819 90 90 9[illegible]	86.0478 128.319 138.489 90 90 90	85.3877 127.408 137.749 90 90 90
Total reflections	3758835 (201888)	713922 (70331)	974131 (95425)
Unique reflections	306646 (28922)	56719 (5572)	78479 (7775)
Multiplicity	12.3 (7.0)	12.6 (12.6)	12.4 (12.2)
Completeness (%)	99.39 (94.50)	99.99 (100.00)	99.20 (99.28)
Mean I/sigma(I)	18.43 (1.15)	16.23 (12.73)	17.91 (7.63)
Wilson B-factor	16.13	15.31	24.27
R-merge	0.08152 (1.328)	0.1503 (0.195)	0.09786 (0.3545)
R-meas	0.08501 (1.435)	0.1567 (0.2034)	0.1021 (0.3707)
R-pim	0.02381 (0.5304)	0.04388 (0.05732)	0.02853 (0.1065)
CC*	1.000 (0.826)	0.998 (0.996)	0.999 (0.997)
Anom Completeness	n/a	100	99.3
Anom Multiplicity	n/a	6.5	6.5
**Refinement**			
Reflections used in refinement	306627 (28914)	56716 (5572)	78415 (7770)
Reflections used for R-free	2001 (187)	1990 (209)	2007 (197)
R-work	0.1533 (0.2931)	0.1397 (0.1538)	0.1494 (0.1943)
R-free	0.1661 (0.3066)	0.1985 (0.2409)	0.1916 (0.2415)
CC(work)	0.973 (0.753)	0.967 (0.959)	0.964 (0.891)
CC(free)	0.976 (0.722)	0.923 (0.873)	0.962 (0.886)
Number of non-hydrogen atoms	13049	12296	12352
macromolecules	11530	11468	11488
ligands	28	38	28
solvent	1491	790	836
Protein residues	1405	1400	1400
RMS(bonds)	0.007	0.008	0.008
RMS(angles)	1.23	0.87	0.85
Ramachandran favored (%)	98.5	98.13	97.27
Ramachandran allowed (%)	1.5	1.8	2.44
Ramachandran outliers (%)	0	0.07	0.29
Rotamer outliers (%)	0.25	0.34	0.51
Clashscore	1.25	3.41	2.65
Average B-factor	21.69	16.64	26.11
macromolecules	20.42	16.5	25.85
ligands	23.41	31.15	31.61
solvent	31.48	17.87	29.42

Table S4. X-ray crystallography data collection and refinement statistics. Numbers in parenthesis correspond to statistics of the highest resolution shell.

	ARO from *A. carboxidovorans*	ARO from *C. firmus*	ARO from *G. sp. Y412MC10*
PDB ID	8SM8	8SM9	8SM7
Wavelength (Å)	1.738	1.738	1.738
Resolution range (Å)	77.41 - 2.0 (2.072 - 2.0)	60.04 - 2.3 (2.382 - 2.3)	82.78 - 2.2 (2.279 - 2.2)
Space group	P 1 21 1	P 1 21 1	P 41 21 2
Unit cell	63.93 154.82 74.95 90 104.93 90	12.818 67.8799 138.508 90 111.74 9[illegible]	127.369 127.369 217.84 90 90 90
Total reflections	796939 (35285)	496671 (46455)	888833 (29415)
Unique reflections	87656 (5715)	82002 (7943)	89804 (7833)
Multiplicity	9.1 (6.0)	6.1 (5.8)	9.9 (3.8)
Completeness (%)	91.31 (59.63)	93.98 (91.13)	98.23 (86.48)
Mean I/sigma(I)	6.70 (0.78)	3.28 (0.68)	7.55 (0.71)
Wilson B-factor	18.72	46.54	40.95
R-merge	0.6088 (2.241)	0.2947 (2.05)	0.1853 (1.501)
R-meas	0.6441 (2.443)	0.3236 (2.265)	0.195 (1.75)
R-pim	0.2055 (0.9472)	0.1312 (0.9431)	0.05892 (0.8554)
CC*	0.968 (0.531)	0.989 (0.815)	0.999 (0.741)
Anom Completeness	n/a	n/a	n/a
Anom Multiplicity	n/a	n/a	n/a
**Refinement**			
Reflections used in refinement	86485 (5649)	81724 (7855)	89703 (7775)
Reflections used for R-free	1995 (128)	1992 (187)	1996 (171)
R-work	0.1918 (0.3481)	0.2030 (0.4000)	0.1986 (0.3916)
R-free	0.2503 (0.4117)	0.2509 (0.4087)	0.2421 (0.4391)
CC(work)	0.892 (0.376)	0.957 (0.533)	0.960 (0.407)
CC(free)	0.887 (0.288)	0.935 (0.485)	0.944 (0.426)
Number of non-hydrogen atoms	11821	11527	11355
macromolecules	11055	11268	10874
ligands	30	8	11
solvent	736	251	470
Protein residues	1376	1376	1388
RMS(bonds)	0.008	0.009	0.008
RMS(angles)	1.04	1.07	1.01
Ramachandran favored (%)	96.93	93.93	94.42
Ramachandran allowed (%)	2.63	5.85	5.29
Ramachandran outliers (%)	0.44	0.22	0.29
Rotamer outliers (%)	0.94	2.12	1.24
Clashscore	4.09	6.97	7.37
Average B-factor	20.21	52.29	48.01
macromolecules	20.02	52.39	48.15
ligands	27.53	59.06	53.49
solvent	22.78	47.37	44.76

Table S4 (continued). X-ray crystallography data collection and refinement statistics. Numbers in parenthesis correspond to statistics of the highest resolution shell.

A0A158JIL1	A0A562RDP6	L9X416	A0A1X2C793	L0IYS5	A0A2D5V3K2	A0A537Y9B3	W6R0B5
A0A1E3Z720	A0A5C8C8Q3	L9XLM0	A0A1X2C8U3	L0IXX4	A0A2E0WU70	A0A537MUS8	A0A6I4PNC2
A0A1H3IEY6	A0A5C8CC54	M0C9C5	A0A1X2CRN1	A0A6N7IKQ7	A0A2E1XQ72	A0A537MTW8	A0A6I4PN86
A0A1H3ID88	A0A5D3JWL9	V4XQY3	A0A1X2CS03	A0A6N7IJ65	A0A2E3Y1C3	A0A537N2G0	A0A6I2FDD6
A0A2R6CG35	A0A5D3JYL3	A0A0M9DJD1	A0A1X2DE14	A0A534Z9I2	A0A2E4V2G4	A0A537N3C9	A0A7J9Z6C1
A0A2R6GWF5	A0A5S4WMM9	A0A0D5NKH7	A0A1X2DDE6	A0A6N7HWK0	A0A2E5H9G2	A0A537Q9G3	A0A8B0S4B6
A0A2R6J6U0	A0A5S4WJG4	A0A0U2W8S0	A0A240B892	A0A6N7HU17	A0A2E7CUK5	A0A537Q9F2	A0A8B0S4Y0
A0A498G0V9	A0A6G6YY05	A0A1R1CCF3	A0A1X2DRL6	A0A8F9ZNJ6	A0A3R7V1L4	A0A537QFN5	A0A7V9K917
A0A498G106	A0A6G6YZ88	A0A3S0W4Z3	A0A1X2E8E0	A0A8F9ZN00	A0A2E0S1W4	A0A537QED6	A0A7V9N5G6
A0A7D5KAJ1	A0A6P1BL93	A0A4Q2HVW5	A0A1X2E8B0	A0A2S9QLD0	A0A7J4URL5	A0A537QQ50	D3F9T7
A0A1H6G4Y1	A0A6P1BMX6	A0A5C4T467	A0A1X2FL56	A0A2S9QLC6	A0A231HAM8	A0A537R229	A0A538LEN7
A0A2R6IFY5	A0A6P1RL45	A0A7C1XFW4	A0A1X2FKQ0	A0A1H3K458	A0A231HA94	A0A537SF47	A0A7W0YV51
A0A2R6IFX0	A0A6P1RH12	A0A7C2GM70	A0A1X2LB36	A0A0N0UA77	A0A024K5M3	A0A537SF41	A0A7Y6CDU3
A0A2R6J2Y9	A0A7L8VM90	A0A7T6Z3Z8	A0A1X2LB27	A0A1H8WA60	A0A024K4R1	A0A537T156	A0A6L7U5N8
A0A4S3TMD5	A0A7L8VS30	A0A7T7CG02	A0A1Z4EMZ8	A0A256IQ23	A0A049DU55	A0A537T134	A0A6N8W3W2
A0A7J9SJ38	A0A7S7QHU9	A0A4Q7V3F7	A0A1Z4EN00	A0A256J2I3	A0A049DY60	A0A537UBK8	A0A845C2D9
A0A7J9SIR5	A0A7S7Q5R5	A0A1Q8JJS4	A0A220Y7P0	A0A2G1WEX4	A0A051TRE2	A0A560EB29	A0A6N8W8K5
M0NJV8	A0A7S7QL74	A0A1Q8JJW1	A0A2A3A207	A0A2R6JMA0	A0A051TRP8	A0A560EB27	A0A2E5HWU8
V4XGM2	A0A7S7QLC2	A0A2N3Y0S1	A0A2A3A1T5	A0A482Y6F5	A0A081I0Q1	A0A560KDE7	A0A845CX66
A0A1I0XJI1	A0A7S7TS16	A0A2N3Y0T4	A0A2A3LAC0	A0A4D6HRG6	A0A081I0Q2	A0A560KK37	A0A845DJQ6
A0A1I1W340	A0A7S7TS95	A0A4R1HYI5	A0A2A3LA31	A0A4V5ZN93	A0A0H3A1M1	A0A560KY59	A0A7C3JLQ3
A0A4Y9ABN8	A0A7S7ZEP6	A0A5N0ULK3	A0A2A7NEI2	A0A521D0C8	A0A0H2ZTN1	A0A560KY03	A0A7C6D2R0
A0A5S3QIY4	A0A7S7UHM1	A0A5N0UL04	A0A2A7NE82	A0A6B9Y1V3	A0A0I9TKU6	A0A562F7F3	A0A7K4AED7
A0A7H8S6Y5	A0A7S7UY26	A0A6H1RCA3	A0A2R5HBJ3	D3SWT3	A0A0I9TPV6	A0A562F7L4	A0A7C7I9S7
A0A1Q4B9V8	A0A7S7ZLX5	A0A6H1RDX8	A0A2R5HAH4	L9WAX5	A0A0M2HBX9	A0A562F9N7	A0A7J9WL75
A0A1H4WMB3	A0A7S9D4T4	A0A840QDE3	A0A2T2ZAG5	M0PT11	A0A0M2HI72	A0A562F9E6	A0A7J9WLR3
A0A2E4W3E5	A0A7S9D619	A0A840QCX4	A0A2T2ZAG0	A0A7D5L9C8	A0A0Q2Q9A1	A0A562LH99	A0A7J9VNL9
A0A381TZZ5	A0A7S9CUH3	A0A852VVA6	A0A2S8C3T2	A0A1H6I8X0	A0A0Q2Q9M0	A0A562LHD7	A0A2M9U0L8
A0A1Z8V6Z7	A0A7S9GTZ5	A0A852W076	A0A2S8C473	A0A2R6G943	A0A0U0W9C2	A0A562LHC1	A0A2N5KRX7
A0A1Z8SAB4	A0A7T7Y056	A0A6G8Q523	A0A895GEM0	G2MNY3	A0A0U0WA13	A0A562RD27	A0A356LM15
A0A2D6SJW9	A0A7T7Y0F6	A0A6G8Q552	A0A895GEP7	A0A1H9NYU5	A0A0U1CWE1	A0A562RE26	A0A328XTX4
A0A2D7CS22	A0A7T7YZP6	A0A535E9W3	A0A2U3NH72	A0A482XZ74	A0A0U1CWE5	A0A5B6TQ41	A0A2V2ZMB2
A0A2D9SDQ8	A0A7T7YZB2	A0A535E990	A0A2U3NGW8	M0A0C5	A0A132TBH1	A0A5B6TPZ9	A0A562JSL7
A0A2E2YIQ3	A0A7W6SIV8	A0A3D9ZM82	A0A2U3NSW1	V4Y1Q6	A0A132TCC7	A0A5C8KX96	A0A2R4XGM7
A0A2E5L400	A0A7W6SIW4	A0A3D9ZN73	A0A2U3NSV3	A0A2E0XGQ4	A0A163Y0B9	A0A5D3KW93	A0A437MLW1
A0A2E7DGL5	A0A7Y4GV59	A0A5B8TZK6	A0A2Z5YLM5	A0A2D6S0M0	A0A162DY35	A0A5D3L1N9	A0A7V9MJ48
A0A2E7DGM6	A0A7Y4GW91	A0A6F8YVB8	A0A2Z5YMP4	A0A2E0XR28	A0A172UL75	A0A5D9CIP3	A0A838JWJ9
A0A2E9UYS1	A0A7Y4M3M8	A0A6F8YUP9	A0A318HCB5	A0A7C7LIY6	A0A172UM47	A0A5D9CGT4	A0A7X1PGC1
A0A6N7FBV6	A0A7Y4M418	A0A543DRG7	A0A318HCI8	A0A2E9MAS7	A0A178LX83	A0A5E8VB00	A0A7X1PBE6
A0A1Q4BMQ3	A0A7Y6SUV9	A0A0V8JC31	A0A329LDF5	A0A2E3YAQ2	A0A178LXP8	A0A5E8VA41	A0A2V7B8A7
A0A1H4PGT3	A0A7Y6T2Y7	A0A0V8JBT5	A0A329L327	A0A2E4MZC0	A0A1A0JDI3	A0A2U8P2R3	A0A1Q6Z2S7
A0A2S6QHC0	A0A7Y6T2X6	A0A2E8WJU5	A0A329MDV6	A0A2E8W9Y9	A0A1A0JE27	A0A5H2YK36	A0A2V6VBV9
A0A2S6QHA1	A0A7Y6SPY4	A0A3B8MBR4	A0A329MAC5	A0A3B8MTU2	A0A1A0LFN2	A0A5P6P7K7	A0A2V7DDH4
A0A2E3ZK23	A0A7Y6SPT7	A0A3B8MBT7	A0A367A5V4	A0A3D2RKF6	A0A1A0LDW8	A0A5P6P7Q0	A0A1H6I5D5
A0A2E5V2R5	A0A7Z0Q8J7	A0A3S1BCH4	A0A367A5W3	A0A127EXZ1	A0A1A0MFW0	A0A5S4W6C2	A0A1H3HLN8
A0A2E6GJ82	A0A7Z0Q8H8	A0A433HWZ4	A0A370GMW9	A0A1X7FTD3	A0A1A0MFX4	A0A5S4WJF4	A0A6B9Y2K4
A0A2E7I253	A0A7Z2SJ85	A0A3S0UJ82	A0A370GPA7	A0A068SY84	A0A1A0TAR6	A0A5S4YQC5	W0JXL3
A0A2E7PJT8	A0A7Z2SND2	A0A496B8D8	A0A375YPR5	A0A068TEU8	A0A1A0TB32	A0A5S4YRE1	A0A1H6JUC6
A0A2E7R0V1	A0A837C4N1	A0A496B8I1	A0A375YPT9	A0A095WTX5	A0A1A0UFU8	A0A6A1TUD2	A0A1I6TMJ7
A0A2E8YSQ7	A0A837C446	A0A4Q2HUX4	A0A386UER8	A0A0D0NJ09	A0A1A0UH71	A0A6A1TSZ4	A0A2R6ISQ8
A0A4V2YGR6	A0A844ST47	A0A4Q2HVH1	A0A386UB81	A0A0Q7WQY3	A0A1A0VG25	A0A6G6YXE7	A0A6B0SCB4
A0A5C4J8E4	A0A844SH77	A0A540VG70	A0A3N1CWH8	A0A0T7FXN2	A0A1A0VG33	A0A6G6YXN1	A0A8A2VAI2
A0A5D0NDP5	A0A850IZ90	A0A6B0VZU8	A0A3N1CWF4	A0A0T7GGB2	A0A1A1W1X1	A0A6G6Z0R5	L9WRI3
A0A1H1I9A3	A0A850J2R1	A0A845CW83	A0A3S4TZU2	A0A1H2BNS1	A0A1A1W0F8	A0A6G6YZJ1	L9XJX2
A0A1H4N763	A0A8I1Y088	A0A6B0XUG6	A0A448IWT6	A0A1M6U253	A0A1A1WAU9	A0A6G8TXS0	L9UVX5
A0A1H4ZR69	H5YKN0	A0A6L7V379	A0A064CLU6	A0A2T7URW8	A0A1A1WB77	A0A6G8TXC9	A0A1H6BFY4
A0A2D9M3Q9	H5YKN1	A0A6B1GU62	A0A064CNS6	A0A3L7AIW6	A0A1A1YIW0	A0A6L5FRY4	A0A1H6JSS3
A0A2E6NH10	I2QHU3	A0A845C1L7	A0A0B2XVT3	A0A4U6BJ30	A0A1A1YIW3	A0A6L5FPR4	A0A1H6JYE3
A0A381U2L4	I2QHU4	A0A6L7QB08	A0A0B2Y894	A0A525JX76	A0A1A1ZS99	A0A6N7GPP6	A0A1H8UDH3
A0A3D4B8E6	J2VIN1	A0A6L7Q6Q4	A0A0D1L8E8	A0A537XNF6	A0A1A1ZS16	A0A6N7GPT4	A0A498F621
A0A3N5MKK9	J3HUE5	A0A6L7U1D4	A0A0D1LMR9	A0A537PXA7	A0A1A2AX08	A0A6P1BHX4	A0A498G8S1
A0A536XL16	K8NTY1	A0A6L7U321	A0A0E3WDN5	A0A5C7SKJ0	A0A1A2AXV8	A0A6P1BH95	A0A6G8TEG9
A0A6L5FFG7	K8NWY3	A0A6N8VMG2	A0A0E4CQG1	A0A6A1TK49	A0A1A2DLG2	A0A6P1QX72	A0A7D5KAK7
A0A1Q7BLH1	Q89D35	A0A6N8VKN2	A0A0G3ITB3	A0A6N7GN42	A0A1A2DK30	A0A6P1QWJ2	A0A7D5LDC0
A0A537TEU4	Q89D34	A0A6N8Y4V1	A0A0G3IU94	A0A840MZ27	A0A1A2DQT1	A0A7C2QT68	A0A7J9SMQ8
A0A6N7FBC0	U1GY64	A0A6N8XW54	A0A0H5RX53	A0A8I1RTR5	A0A1A2DPL3	A0A7C2QUP2	A0A830DVL8
A0A537VJ64	U1HCC1	A0A7X3RPW1	A0A0H5RGP5	W6WQ50	A0A1A2EV89	A0A7C9VI65	D4GQB6
A0A7W9FM05	A0A1H4Y060	A0A7X4BN19	A0A0J6Y6I9	A0A2A7WVK1	A0A1A2DZW9	A0A7L8VH11	M0IHA2
A0A0N9ICA6	A0A1H4Y0D2	A0A7X4BMP1	A0A0J6VGA6	A0A2A7X2N7	A0A1A2GD82	A0A7L8VGX0	V4YFL6
A0A178X634	A0A1Q4B8L5	A0A7X4FDM7	A0A0J6WDK8	A0A2C2VGR5	A0A1A2GD19	A0A7S7QGX7	B5I3L3
A0A1I5HVH1	A0A1Q4B8X7	A0A7X4FE69	A0A0J6WBM3	A0A2E0S3C4	A0A1A2GNL4	A0A7S7QHL5	A0A7K2PE83
A0A2K8M2W4	A0A5S4VVH3	A0A845C1D4	A0A0K0XG92	A0A3N6N0L8	A0A1A2GM37	A0A7S7QKG8	U5YQT4
A0A7W3VWL4	A0A5S4VYC2	A0A845C636	A0A0K0XG51	A0A7J4SPQ4	A0A1W9Y980	A0A7S7QKE9	B6A5M1
A0A845J550	A0A1L7ALY1	A0A7J9VJ14	A0A0M2ZLL5	A0A7J4UQI8	A0A1W9Y9I6	A0A7S7QVB4	A0A859QEI9
A0A8B0S6C0	A0A1L7ALX1	A0A7J9VL75	A0A1X0D5G3	A0A7J4URF5	A0A1A2J249	A0A7S7TNB1	E7QUX1
A0A2S9ADG8	A0A4V1T317	A0A4Q3G9Q8	A0A0N9XGP1	A0A7J4SL13	A0A1A2J165	A0A7S7T295	A0A1M6NEV3
A0A0F0L1R6	A0A4Q3FDT2	A0A4Q3GBX7	A0A0Q8ZMB0	A0A3N6LNW2	A0A1A2MSU1	A0A7S7T4C2	F2L731
A0A0F0LR51	A0A1Y5LIZ8	A0A838SSW0	A0A0Q8ZKC9	A0A290Q3R4	A0A1A2MSU6	A0A7S7T6C7	A0A535EDB3
A0A142MDZ6	A0A1Y5LG18	A0A8I1PDM3	A0A0Q9K9P8	A0A290QG37	A0A1A2NZ98	A0A7S7T8R6	F4CY60
A0A142ME00	A0A6N7CMB8	A0A1H1QKM4	A0A0Q9JZZ4	A0A2T6DUE0	A0A1A2PF28	A0A7S7UWA3	A0A431NFZ2
A0A259SIR6	A0A6N7CM05	A0A1H1QKN5	A0A0U1DX08	A0A2T6DUD7	A0A1A2PEU8	A0A7S7ZM72	A0A434SJ79
A0A2S8ZNZ7	A0A897DHZ4	A0A0B0I692	A0A1X1SJH2	A0A2E7JWE6	A0A1A2PPY4	A0A7S7TR33	W0JXZ8
A0A2T7UT90	A0A897DEG7	A0A0B0ID52	A0A100WSA7	A0A6B0XDL4	A0A1A2PPY9	A0A7S7TR68	A0A1H3KR99
A0A2T7UT14	A0A1X6YC48	A0A0U2U9T5	A0A100WS36	A0A7X3TPM2	A0A1A2QGL4	A0A7S7ZEU1	A0A1H6AGY1
A0A316H9L1	A0A1X6YAG2	A0A0U2U9H7	A0A117IHC0	A0A6L7QIB7	A0A1A2QFR8	A0A7S7UH51	A0A1H6BA07
A0A316HEK7	A0A127CNJ7	A0A1H1R589	A0A124E5R3	A0A836UIZ8	A0A1A2RDA0	A0A7S8AAH0	A0A498G491
A0A381IM77	A0A127CNU9	A0A1H1R7W7	A0A100XCE0	A0A836U6K8	A0A1A2REG3	A0A7S8AAG9	A0A498G9G2
A0A381INV2	A0A2A4XVD2	A0A1I3A8M6	A0A100XCE5	A0A836XGR8	A0A1A2TKV5	A0A7S9CS16	A0A6A9SPJ3
A0A5B9DIQ4	A0A2A4XW04	A0A1I3A9B5	A0A100ZVL7	A0A2Z4AGE7	A0A1A2TH34	A0A7S9CRV9	A0A6B0SDP3

A0A5B9DIY3	A0A2A4YPI7	A0A1Q9RZR6	A0A124EML6	A0A2D6CZR2	A0A1A2V7L1	A0A7S9GZW8	A0A6G8TEB5
A0A6I2LHS1	A0A2A4YQS5	A0A2E6HXB0	A0A101A1X5	A0A535EB79	A0A1A2V739	A0A7S9D506	A0A7D5H1W7
A0A6I2LBW7	A0A2D8K973	A0A2E6HX71	A0A101A1L7	A0A7J9X5H5	A0A1A2VES9	A0A7T7XXE1	A0A830FTY3
A0A7K1TV20	A0A2D8K8Y2	A0A4Q7X042	A0A101A5Z9	A0A1H0AD20	A0A1A2VE99	A0A7T8BJ35	A0A830G906
A0A7L8S4E2	A0A2U2CD25	A0A4Q7X0S4	A0A101A5W8	A0A1G8HRQ7	A0A1A2W8H9	A0A7T7Y7L1	A0A830GCU9
A0A7L8S4E1	A0A2U2CDA9	A0A4R0JHF8	A0A101AU25	A0A6G1X1K4	A0A1A2W9H9	A0A7T7Y7J2	A0A8A2VC12
A0A7W3Q678	A0A438AHC9	A0A4R0JFI1	A0A117JS91	A0A7J9X101	A0A1A2WMF3	A0A7T7Z246	A0A8A2V8C2
A0A7W3KSW1	A0A438AHI9	A0A4R0KSY0	A0A117JVX9	A0A6I1R8P4	A0A1A2WKG0	A0A7T7Z1R4	L9WN84
A0A8E1WHN8	A0A5C4JXD3	A0A4R0KWN1	A0A117JVY3	A0A836XHE1	A0A1A2WPU1	A0A7T8A3Q4	L9WP05
A0A8E1WGV2	A0A5C4JWQ8	A0A4R3LTJ3	A0A101BIA7	A0A329EIS2	A0A1A2WRC5	A0A7T8A2G0	L9XKU6
A0A6L9IP60	A0A7T7KLH3	A0A4R3LTI7	A0A101BIA6	A0A366Y165	A0A1A2NU89	A0A7W0TY72	M0CRW7
A0A0Q6X8P5	A0A7T7HKC3	A0A4R7ZU50	A0A117I822	A0A3M8C6C1	A0A1A2XG79	A0A7W0ZPN0	M0LUN0
A0A0Q6WZR2	A0A7W6PC75	A0A4R8C2V5	A0A117I831	A0A3M8CVR4	A0A1A2YEZ5	A0A7W6STY1	W2EMD6
A0A2U3PCE3	A0A7W6PDA8	A0A4R8C149	A0A117JAV0	A0A3M8CV62	A0A1A2YEC8	A0A7W6SSD2	A0A1C4WKP2
A0A2U3PCE8	A0A3E0VKK7	A0A4U3M1Y5	A0A100ZEW2	A0A6H1NYY8	A0A1A2YYD0	A0A7W6TUX9	A0A1H2B560
A0A5B0VS64	A0A3E0VKV1	A0A4U3M3N4	A0A117JBL4	V6MEI2	A0A1A2YZ37	A0A7W6TUZ7	A0A1H9JIR1
A0A5B0VQS9	A0A1G9HTE1	A0A521TKT7	A0A117JBK8	A0A7G8TV23	A0A1A2ZLC7	A0A7W6X4Z3	A0A2T0MR73
Q0RW58	A0A1G9HTN5	A0A521TKT4	A0A101AQG6	A0A1N7FT18	A0A1A2ZJL3	A0A7W6X4B0	A0A2W2C3F1
Q0RW56	A0A4V3IPE1	A0A521TNU7	A0A101AQ91	A0A1G8EE52	A0A1A3CBK2	A0A7W9FQW6	A0A4R4N1S9
A0A1Y5KNW9	A0A4R8ZH18	A0A521TNV5	A0A101BCM9	A0A1I4C321	A0A1A3CA00	A0A7W9FQX5	A0A4R4RNJ2
A0A1Y5KWP6	A0A4T2C180	A0A535Y0N6	A0A117JYF8	A0A2B2BY47	A0A1A3CPQ4	A0A7Y4GUF9	A0A4R4VZQ6
A0A8A3QX11	A0A4T2C1T8	A0A535Y0M6	A0A132PQ89	A0A3M8CRU0	A0A1A3CSD6	A0A7Y4GTS1	A0A4R5FX23
A0A8A3QW31	A0A542IPP1	A0A536DM77	A0A132PPZ0	A0A3M8CW48	A0A1A3EED4	A0A7Y4M1H2	A0A5C4VDW5
A0A8F8S1V0	A0A542IPM5	A0A536DMH3	A0A1A0KWZ4	A0A4R5VL45	A0A1A3EEU0	A0A7Y4M1S7	A0A5C4VDW5
A0A8F8WUY1	A0A7D5KP59	A0A561T0I3	A0A1A0KVE7	A0A4U0FD42	A0A1A3EI19	A0A7Y6VZL3	A0A5R8MWX1
A0A3N2GP79	A0A1D2QKF7	A0A561T0G9	A0A1A0Q480	A0A5J5HBP3	A0A1A3EGR7	A0A7Y6VZX4	A0A6G6WDL7
A0A3N2GP77	A0A1H6G865	A0A6I5VS36	A0A1A0Q6R1	A0A890FB15	A0A1A3EYN2	A0A7Y6T1Y7	A0A6I3JCE5
A0A8E1W785	A0A2R6J8A8	A0A6I5VPM8	A0A1A0QJ42	K6D546	A0A1A3EYV3	A0A7Y6T223	A0A6J4LIP7
A0A8E1W6A5	A0A4S3TNY5	A0A6J4V716	A0A1A0QK89	A0A1I3R8K1	A0A1A3G521	A0A7Y9RMW2	A0A7J9Y4Y3
A0A024K2V1	A0A6N2CQF2	A0A6J4VG21	A0A1A0SJH1	A0A2G5WHE2	A0A1A3G413	A0A7Y9RP43	A0A510HM36
A0A024K202	A0A1K1PUC7	A0A6L7UDD2	A0A1A0SKC2	A0A2G5W2K8	A0A853M2G6	A0A7Y9Z5U7	A0A6N7HX24
A0A0E3WD92	A0A0Q9N3I1	A0A6L7UDK6	A0A1A0UL75	A0A2G5XLK7	A0A853M6T7	A0A7Y9Z8C6	A0A6N7I8I9
A0A0E4H011	A0A0U2ILT9	A0A6M6JPS2	A0A1A0UL72	A0A3T1DD03	A0A1A3IIV9	A0A7Z0Q8V6	A0A7J9XDJ7
A0A163W679	A0A0U2W203	A0A6M6JRS7	A0A1A0WNQ2	A0A559JWG8	A0A1A3IIU4	A0A7Z0Q9X4	A0A838JRV7
A0A163W699	A0A172TNG9	A0A6N7HXM7	A0A1A0WN06	A0A1M6T6Z6	A0A1A3JYG0	A0A7Z2PS24	Q1AYM1
A0A1A0KRG5	A0A197ZZY7	A0A6N7HUT2	A0A1A1X807	A0A1M6T7I9	A0A1A3K1I4	A0A7Z2PRU4	A0A2N5GGD3
A0A1A0KQW7	A0A1R1CBA4	A0A6N7IB48	A0A1A1X6V1	A0A1Q4E6B1	A0A1A3LS16	A0A837C5A3	A0A3A0BU93
A0A1A0LVX4	A0A1Y5KQM8	A0A6N7IBS9	A0A1A0TF50	A0A1Q4E5X5	A0A1A3LV58	A0A837C4Y6	A0A535I8X8
A0A1A0LXQ6	A0A2B2BY04	A0A7J9X938	A0A1A0TDZ5	A0A292YVR7	A0A1A3NEL9	A0A839UL63	A0A7V8WJX7
A0A1A2DUF4	A0A3B0CJV8	A0A7J9X8L0	A0A1A2DI07	A0A292YXM6	A0A1A3NCD8	A0A839ULX9	A0A7V9KIG5
A0A1A2DTG5	A0A430JKU3	A0A7J9XQR6	A0A1A2DGP0	A0A431NFJ5	A0A1A3QPI6	A0A840GXQ5	A0A7W0NM91
A0A1A2G4N0	A0A4Q3A6S0	A0A848DJ30	A0A1A2LG72	A0A431NF71	A0A1A3QND3	A0A840H6B0	A0A7W1HTR3
A0A1A2G6S6	A0A4Q9DNQ7	A0A848DJ48	A0A1A2LET3	A0A4Q3PT18	A0A1A3KD72	A0A840HKV9	A0A4P6JP68
A0A1A2H9Z9	A0A4Q9DJ64	A0A852ZSJ8	A0A1A2LSB7	A0A511DAF5	A0A1A3KBS2	A0A840HL84	A0A3D0S354
A0A1A2HA18	A0A4Q9DHC3	A0A852ZTQ6	A0A1A2LTC9	A0A511D9Y2	A0A1A3TLD0	A0A840XVU6	A0A3S1B2G0
A0A1A2MJ43	A0A4Q9DQT0	A0A8F9ZM67	A0A1A2LZK7	A0A6M6JQS4	A0A1A3TLJ2	A0A840Y5L2	A0A7J4SMZ1
A0A1A2MJ29	A0A5C4SW66	A0A8F9ZLT3	A0A1A2M0B4	A0A6M6JNJ9	A0A1A3TNF3	A0A844S306	A0A7W0UK52
A0A1A2P403	A0A5C4T1B0	A0A6G8QEV5	A0A1A2Z0H1	A0A7G7MCN4	A0A1A3TNH9	A0A844RZT6	A0A1Q3V3A8
A0A1A2P427	A0A5C4T0A3	A0A6G8Q4V4	A0A1A2Z0Z8	A0A7G7MCN5	A0A1A3U4G3	A0A844SLY4	A0A1Q3V1W0
A0A1A2PL56	A0A5C4T569	A0A402C2Q3	A0A1A3DHD8	A0A7J9Z823	A0A1A3U4L1	A0A844SRL9	A0A257PYP1
A0A1A2PL28	A0A5C4T4H2	A0A402C2V4	A0A1A3DGS2	A0A7J9Z832	A0A1A3C330	A0A844SZQ2	A0A257PXW5
A0A1A2V9B3	A0A5C4TBL5	A0A439MRZ4	A0A1A3KST5	A0A8I1PDT5	A0A1A3C1E3	A0A844SYH8	A0A2S6N1I3
A0A1A2VBC6	A0A5C4TAG8	A0A440KRE0	A0A1A3KRA5	A0A8I1TKU9	A0A1A6BEQ3	A0A844TIY2	A0A2S6N1H4
A0A1A2VIP0	A0A5R9G5L2	A0A440KRE6	A0A1A3PJ98	F4CZ80	A0A1A6BEE9	A0A844THR7	A0A2V3UDR8
A0A1A2VGG7	A0A5R9GCF6	A0A529UIC3	A0A1A3PI79	F4CZ84	A0A1B7UCW0	A0A845IXC0	A0A2V3UEL9
A0A1A2WMK6	A0A7W0D2M2	A0A529Y653	A0A1A3T2Y2	A0A7K0P9X9	A0A1B7UDC9	A0A845IPP0	A0A354V7J2
A0A1A2WL07	A0A858RF10	A0A531Z9S3	A0A1A3T2T2	A0A543KRR8	A0A1B8SE92	A0A848EE72	A0A354V7P3
A0A1A2ZPF9	A0A2T6DBW4	A0A531Z9H6	A0A1A3T840	A0A6J7FUC8	A0A1B8SEE7	A0A848EC76	A0A4V3NCA4
A0A1A2ZQ74	A0A7G9S5C8	A0A5S4VW82	A0A1A3T843	A0A1F6CZ79	A0A1B9DAN1	A0A849N8T9	A0A4S0W165
A0A1A3A8Q6	A0A7G9S5C9	A0A5S4VV21	A0A1E3R7K3	A0A2W5ZQ37	A0A1B9DAU3	A0A849N824	A0A504ANF9
A0A1A3A7F8	A0A076N111	H5YAK8	A0A1E3R629	A0A399ZQW5	A0A1E3RJ17	A0A850I2Q6	A0A504AZV8
A0A1A3C5Z4	A0A076N484	H5YAK9	A0A1E3SWM2	A0A402BDT6	A0A1E3RJ13	A0A850IAG1	A0A6B9FV34
A0A1A3C560	A0A0P0SAK9	X5PS67	A0A375YXU3	A0A2E0KZ06	A0A1E3SJ57	A0A850ISR8	A0A6B9G1A4
A0A1A3DRN6	A0A0P0SJ55	X5PTA3	A0A1E7Y6Z0	A0A2E0KXI2	A0A1E3SJ01	A0A850J095	A0A1I2LC77
A0A1A2GEC2	A0A0Q6GJW2	A0A6J4VSL9	A0A1E7Y6S9	A0A2E0L7N6	A0A1E3T8Z2	A0A850IZL4	A0A6J7KEN3
A0A1A3QTA7	A0A0Q6GL90	A0A1E4LEQ0	A0A1G6WDI4	A0A7Y5Q3W4	A0A1E3T981	A0A850J359	A0A7K0PJN4
A0A1A3QVE9	A0A0Q6JPX4	A0A1H0BBE9	A0A1G6WE09	A0A0B0HPP6	A0A1E8Q4L9	A0A859R1E0	A0A1M3NV50
A0A1R0U2Q9	A0A0Q6JXB5	A0A1H0BBI8	A0A1H6KKZ4	A0A0C2V692	A0A1E8Q377	A0A859QRS2	A0A127EWB0
A0A1R0U288	A0A1Q8JUX5	A0A1H2B5Y0	A0A1H6KAI7	A0A1R1DVG5	A0A1H4APR0	A0A8E8XU88	A0A127EUQ0
A0A1R0VFW8	A0A1Q8JUY1	A0A1H2B677	A0A1I2SQ12	A0A259T8U5	A0A1H4APE3	A0A8E8XSU5	A0A1K2HWE6
A0A1R0VFX2	A0A1Q8KJK0	A0A1M3KNL4	A0A1I2SQF1	A0A3D9GBZ9	A0A1M7KH43	A0A8E8YD89	A0A345ZUT8
A0A1S2WM10	A0A1Q8KJJ7	A0A1M3KMW1	A0A1J0U2G4	A0A839U3B6	A0A1M7KH23	A0A8E8YCL4	A0A6N7GN55
A0A1S2WN97	A0A1Y2MLZ2	A0A1Q3YXH6	A0A1J0UGA1	A0A1K1QQ30	A0A1Q4HNA0	A0A8E9CKF9	A0A6N7GQJ0
A0A1V3XHC0	A0A1Y2MLX3	A0A1Q3YXF2	A0A1Q4HKX0	A0A0Q9N0R2	A0A1Q4HNP4	A0A8E8Y2N6	A0A433XKR6
A0A1V3XGQ4	A0A2N3Y134	A0A3A9JID8	A0A1Q4HMA7	A0A172TFK4	A0A1Q4HSJ4	A0A8I1RY37	A0A0Q7TZS7
A0A1W9Y6Z3	A0A2N3Y135	A0A3A9JRZ2	A0A1Q4T8R4	A0A1I1TYQ1	A0A1Q4HSW4	A0A8I1RRJ8	A0A371BAE9
A0A1W9Y746	A0A318LE85	A0A3S0GSF3	A0A1Q4T8Y9	A0A1Y5KGU4	A0A1R0UDR5	A0A8I2C4D4	A0A1P8UST8
A0A1X0D5B2	A0A318LNH1	A0A431NBC6	A0A1Q8Y2X7	A0A430JIK8	A0A1R0UE34	A5EJG9	A0A0B5DWC7
A0A1X0D4S2	A0A365ZFW8	A0A437MER9	A0A1Q8Y389	A0A4V2J492	A0A1R0VEY5	A5EJH0	A0A1G7T5C4
A0A1X0EBU2	A0A365ZF55	A0A437MEQ2	A0A1R0V829	A0A559JHN9	A0A1R0VEX8	H0SDY1	A0A1H0GIR3
A0A1X0EA08	A0A3L7XC48	A0A4R0YAK6	A0A1R0V892	A0A5C4TCJ9	A0A1S1NRP2	H0SDY2	A0A1U7DEG2
A0A1X1UDZ8	A0A3L7XC47	A0A4V2NJS5	A0A1Q9W543	A0A5R9GBA8	A0A1S1NJQ5	H0TM56	A0A225NEB8
A0A1X1UE19	A0A496B7B2	A0A7X3YA39	A0A1S1K507	A0A7X2ZBQ9	A0A1S2WPF6	H0TM57	A0A238JK21
A0A1X2AFW2	A0A496B613	A0A7X3YA63	A0A1W9YYJ1	A0A7X3CRT4	A0A1S2WPK0	H0TVT3	A0A291GET1
A0A1X2AFC2	A0A554QYW7	A0A8I1R3L8	A0A1W9YYR5	F8FQJ3	A0A1V3WUZ1	H0TVT4	A0A2E0IGY0
A0A1X2EAA5	A0A554QYX3	A0A8I1R6J2	A0A1X0B3S2	H6NCP8	A0A1V3X6L9	H5YD41	A0A2M8J4F5
A0A1X2E9P8	A0A6N8WA43	F4CRX1	A0A1X0B2F1	I0BFQ5	A0A1V4Q047	H5YD42	A0A5E7XR34
A0A1Z4EL80	A0A6N8WA26	F4CRX2	A0A1X0BKQ5	A0A367YUD2	A0A1V4Q0G1	I2QM70	A0A5P9BMQ7
A0A1Z4EL67	A0A7I9WN69	A0A2E0H5J0	A0A1X0BKM5	A0A7C7WHP4	A0A1W9ZNT7	I2QM71	A3VMH8

A0A2K4YEM6	A0A7I9WMI1	A0A2E0H5E6	A0A1X0FSZ4	A0A516PVZ3	A0A1W9ZNB6	J3CYU9	Q0FUL9
A0A2K4YEM9	A0A7K2TMI8	A0A2E5L7V2	A0A1X0FTQ2	A0A3C1JQP8	A0A1X0A458	J2WS18	A0A1Z9ESB5
A0A2R5H8V3	A0A7K2TMI9	A0A2E5L7L6	A0A1X0IL30	A0A3B9Z257	A0A1X0A4T3	K8P1P9	A0A1Z9ES83
A0A2R5HEI6	A0A845DDA1	A0A7G5LJ87	A0A1X0IKZ9	A0A3L7Y8R9	A0A1X0BP25	K8PDT9	A0A2D8G6B6
A0A2S9QYR5	A0A845DML9	A0A7G5LJ88	A0A1X1RPD7	A0A4P5V2U0	A0A1X0BMM9	Q89C90	A0A2D8G6D5
A0A2U3NJ60	A0A845D8A9	A0A2D7CP28	A0A1X1RPD4	A0A6L7U2U3	A0A1X0D4U3	Q89C89	A0A2E3K9H3
A0A2U3NJ65	A0A845D679	A0A2D7CP74	A0A1X1V2M9	A0A845CBZ8	A0A1X0D4T0	U1GRB8	A0A2E3K9P6
A0A2U3NW77	A0A897DBV8	A0A1Z8SMU0	A0A1X1V2V3	A0A1Y2MX10	A0A1X0DV00	U1H5X6	A0A2E3ZLE9
A0A2U3NW42	A0A897DGQ0	A0A1Z8SN03	A0A8E4KVZ9	A0A1Y2MX14	A0A1X0DVS0	W3RM40	A0A2E3ZKV8
A0A2U3PCY2	A0A7J9WYB0	A0A2D6SJE2	A0A8E4KY09	A0A076MSM6	A0A1X0E7D6	W3RLZ2	A0A2E5V1W1
A0A2U3PCY8	A0A7C2VLB3	A0A2D6SJE3	A0A1X1XM82	A0A076MT98	A0A1X0E8A5	W6VZT8	A0A2E5V1R4
A0A498Q936	A0A7V9HJT7	A0A2D9MRS5	A0A1X1XMJ7	A0A0M3U9P9	A0A1X0G295	W6W6C1	A0A2E6GJY0
A0A498QVJ7	A0A7V9HJN4	A0A2D9MRQ4	A0A1X2B4W6	A0A0M4R2A4	A0A1X0G2C4	W6VZ68	A0A2E6GJV6
A0A498QXQ6	A0A7W9SL38	A0A2D9YBA0	A0A1X2B4H2	A0A1I3VPY8	A0A1X0GK40	W6VY48	A0A2E7QZH0
A0A653F8U1	A0A831X3D8	A0A2D9YAB5	A0A1X2FFL0	A0A1I3VQA1	A0A1X0H6E6	W6WGN2	A0A2E7QZN8
A0A7G1I8G2	A0A254RG85	A0A2E7V9P9	A0A1X2FEZ2	A0A1Q8L1M2	A0A1X0H6R9	A0A095VL67	A0A2S6QCX7
A0A7I7QGQ2	A0A254RJ93	A0A2E7V9G8	A0A1X2LT18	A0A1Q8L1L8	A0A1X0IGS7	A0A095WT89	A0A2S6QCM6
A0A7I7QGG8	A0A844VZL3	A0A2E9UWJ3	A0A1X2LT57	A0A1Q8LRP2	A0A1X0IGT9	A0A095XMR8	A0A3D2BQ85
U5WLN1	A0A844W8R8	A0A2E9UWJ4	A0A1Y0C1I4	A0A1Q8LRN4	A0A1X0JRT4	A0A095VHH1	A0A3D2BQN5
U5WKY6	A0A4Q7V1L1	A0A840AER6	A0A1Y0C1K2	A0A1Q9SH74	A0A1X0JRZ0	A0A095VIA9	A0A3L7A2F3
X7Y3W3	A0A1Y5LEF9	A0A840AC70	A0A255D771	A0A1G6W0A7	A0A1X0KJF4	A0A0A3XY64	A0A520NWZ0
X7Y2P4	A0A2A3EU86	A0A8A6KKB1	A0A255DBW2	A0A222VRZ6	A0A1X0KJS3	A0A0A3XV44	A0A520NX12
X7ZJP9	A0A6N7CD13	A0A8A6KE74	A0A2C9T7L3	A0A263E1K4	A0A1X0Y9X9	A0A0D1NPR0	A0A523D9H1
X7ZLX1	A0A6P1I6Z3	A0A442JGV3	A0A2C9T712	A0A263E146	A0A1X0Y984	A0A0D7N6K3	A0A537N8X3
D5PGT3	A0A897DIM4	A0A441BXX1	A0A2G8B7N7	A0A292YUV7	A0A1X1QXS5	A0A0D7N5V8	A0A3N7PYS5
D5PGT4	A0A8A6S1P1	A0A528ALM7	A0A7R7TUK7	A0A292YPK9	A0A1X1QXY5	A0A0D7PMZ2	A0A4V6P2U9
A0A0Q2XD86	A0A198ACA3	A0A442M8W4	A0A2K4YGQ5	A0A2V4AN80	A0A1X1RHW9	A0A0D7PM86	A0A525J4M3
A0A0Q2QH67	A0A0Q9MUY6	A0A4Q3DWT0	A0A2K4YGS1	A0A2V4AQ52	A0A1X1RHX8	A0A0D7Q955	A0A849I9R5
A0A0U0W6S7	A0A0Q9QLQ2	A0A4Q3DX13	A0A2M9F897	A0A3N2G8K0	A0A1X1T191	A0A0D7Q5L3	A0A0Q7I795
A0A0U0W762	A0A198AJU7	A0A6N7G0T9	A0A2M9F8A2	A0A3N2G6F5	A0A1X1T164	A0A0E3VTA3	A0A127F425
A0A0U1DDX2	A0A1H0KMI7	A0A6N7FV87	A0A2N8LHB2	A0A431NG05	A0A1X1T963	A0A0E4BM96	A0A127F6N0
A0A0U1DC99	A0A1R1BCH9	A0A1H3LAM4	A0A2N8LH25	A0A431NFF2	A0A1X1T994	A0A1L3FQX3	A0A127F4T6
A0A1A0P0B6	A0A1R1BCK7	A0A1H3LBY8	A0A2S7NE04	A0A4R4VRM3	A0A1X1U3H9	A0A1L3FQS4	A0A1Q7B4G9
A0A1A0P185	A0A1Y5KDI8	A0A2E0IRR9	A0A2S7NDJ3	A0A4R4VQ10	A0A1X1U3T9	A0A1M3PAC4	A0A1Q7BLI5
A0A1A0U9Y9	A0A229UM81	A0A2E0IPR6	A0A2S8KCH5	A0A543GG67	A0A433AP00	A0A1M3PA61	A0A1W6ZZC7
A0A1A0U9V9	A0A2A2QMH7	A0A2E2RRK3	A0A2S8KXJ4	A0A543GG73	A0A3S0RQG1	A0A1M3QA09	A0A537NMN7
A0A1A1WMW1	A0A2A2QNR2	A0A2E2RPM4	A0A2S8KXS4	A0A558A3D0	A0A3S4FTP8	A0A1M3QA15	A0A537P3F7
A0A1A1WPA5	A0A329MTY4	A0A2S3UJY3	A0A2U3P9L9	A0A558A3D1	A0A3S5D022	A0A1M5I164	A0A537TZ11
A0A1A2AZV6	A0A3B0CK98	A0A2S3UJU1	A0A2U3P9L1	A0A561T0Z9	A0A494SZU1	A0A1M5I150	A0A5B8LVT2
A0A1A2AYR7	A0A3G9IKG3	A0A3M2DYK1	A0A2U9PV40	A0A561T127	A0A494T3T4	A0A1M5LIL1	A0A6L5FQS1
A0A1A2IYR7	A0A3G9JF72	M4ZUR3	A0A2U9PV49	A0A5B0J2T6	A0A498PKH4	A0A1M5LIB6	A0A6N7GVD7
A0A1A2J094	A0A4Q9DLF1	M4Z9S8	A0A2V0N9Z0	A0A5C4M5Q6	A0A498PL37	A0A1M5W768	A0A6N7GWS0
A0A1A2M5A4	A0A4Q9DLI8	A0A023XX26	A0A2V0N262	A0A5C4M758	A0A498QDA6	A0A1M5W7B0	A0A7C4TQG9
A0A1A2M3U9	A0A4R5KGL5	A0A023XX05	A0A2X1S7B4	A0A6N7HXZ7	A0A498QCM0	A0A1M5W7K6	A0A7X8TG54
A0A1A2WSW4	A0A5C4SXZ4	A0A068SQH5	A0A2X1S6N4	A0A6N7HY01	A0A498QXM7	A0A1M5W8H3	A0A6L5FEW4
A0A1A2WSF1	A0A5C4SXT8	A0A068SRG6	A0A329LIH9	A0A6P2C8V1	A0A498QZ36	A0A1M5X2V1	A0A1Q4BAX9
A0A1A2YPC6	A0A5C4T8P8	A0A068T924	A0A329LGT0	A0A7X5URP3	A0A4R6K9B7	A0A1M5X3P2	A0A1F3ZIB6
A0A1A2YSG8	A0A5C4TAR2	A0A068T7M4	A0A370D3T1	A0A7X5URR0	A0A4R6K9N6	A0A1M5XR57	A0A1H4V2M8
A0A1A3BHX6	A0A7X2ZB17	A0A0Q5ZS87	A0A370D027	A0A7Y8TMG8	A0A4R8IRG7	A0A1M5XQK2	A0A859QL32
A0A1A3BHV2	A0A6N7I833	A0A0Q5ZRI5	A0A378SPS4	A0A7Y8TLL5	A0A4R8IZB3	A0A1M6RQ12	A0A1H0KEW5
A0A1A3J1A5	A0A6L7JGZ5	A0A0R3BPJ3	A0A378SS84	A0A7Y9J3Q8	A0A502E891	A0A1M6RQ33	A0A4Q2J1L5
A0A1A3DA95	A0A6L7JEV1	A0A0R3BPI9	A0A378SZL6	A0A7Y9J3I8	A0A502E622	A0A1M6XPW7	A0A6N7GQ38
A0A1A3QQU0	A0A7W1LEW8	A0A0R3C9P9	A0A378T0X6	A0A852W0T3	A0A522NM37	A0A1M6XPX2	A0A132T7Y5
A0A1A3KS66	A0A2P8CXF4	A0A0R3CGJ5	A0A378UR24	A0A852W6J2	A0A522NL66	A0A1M6Y9Q7	A0A2D8G2Q9
A0A1A3MUD0	A0A7V9RQZ1	A0A0R3CQL0	A0A378UQS9	A0A852VZN4	A0A545B094	A0A1M6YA19	A0A2D8PPZ5
A0A1A3MS32	A0A7W0Z577	A0A0R3CXQ3	A0A5E8P6I2	A0A852W0Z2	A0A545B0A8	A0A1M7U2J3	A0A2S6Q726
A0A1A3UHV9	A0A838K3F3	A0A0R3E2C3	A0A5E8P899	A0A892P747	A0A557Y1E7	A0A1M7U2V3	A0A520NNQ8
A0A1A3N423	A0A838GZP3	A0A0R3E2B5	A0A3S4S7Z2	A0A892P3Q3	A0A557Y1J0	A0A1Q3K3L9	A0A536DRP6
A0A1A3P3A7	A0A838SVS1	A0A0R3M563	A0A448I3W9	A0A8G0DYA6	A0A5P8ZT87	A0A1Q3UZM9	A0A6G8Q117
A0A1A3P1U9	A0A7C1EYK0	A0A0R3LXE0	A0A439DR27	A0A8F9YI61	A0A5P8ZVG1	A0A1Q4B9A1	A0A843T7D7
A0A1A3Q3X3	A0A3L7YGF0	A0A0R3M2E5	A0A439DR25	A0A8F9ZP96	A0A5Q2MGD5	A0A1Q4B8Q6	A0A542XB56
A0A1A3Q2Z3	A0A7V9KXX8	A0A0R3M6R6	A0A498QET4	A0A8F9ZP44	A0A5Q5BN77	A0A1Q4B9V2	A0A427E2S5
A0A1A6BFU9	A0A158EGK1	A0A0R3MPU7	A0A498QEY5	A0A8I1PB30	A0A5Q5BN27	A0A1Q4B9T3	A0A427E341
A0A1A6BG41	A0A158EGX3	A0A0R3MRI5	A0A4Q7Q319	A0A8I1PAB6	A0A5Q5CJD2	A0A1Q4C2E2	E5XLX3
A0A1B9CJW9	A0A2A6M7U4	A0A0R3NE62	A0A4Q7PZY4	H5WYR2	A0A5Q5CJR7	A0A1Q4C2N8	A0A2A9H0H2
A0A1B9CJT0	A0A2A6M885	A0A0R3NDZ8	A0A4R6HEV6	H5WYR3	A0A5Q6RY94	A0A1Q5RVZ9	A0A4Q7YFW2
A0A1E3T6M5	A0A2L0HA45	A0A0S6UME3	A0A4R6HGG7	A0A4R6VS03	A0A5Q6RYR0	A0A1Q5RW03	A0A255GBF6
A0A1E3T8N9	A0A2L0HA19	A0A0S6UNV7	A0A4Y9MYF2	A0A4V3DB68	A0A653ER97	A0A1Q5S548	A0A7V8RU78
A0A1Q4HUZ7	A0A2U3I209	A0A0T1WWI3	A0A4Y9MXH8	A0A2N9B375	A0A6B9BBT8	A0A1Q5S4Z7	A0A7V8LJ47
A0A1Q4HV81	A0A2U3I210	A0A0T1WXG1	A0A4Z0HYV8	A0A2N9B3A5	A0A6B9BIJ3	A0A1Q5S8Z2	A0A1E4I9K8
A0A1X0IEB2	A0A3D8K7V6	A0A0T7FNM2	A0A4Z0HYC5	A0A429DPU4	A0A6F8YA49	A0A1Q5SFM0	A0A1E4I924
A0A1X0IDU8	A0A3D8K6J4	A0A0T7FNJ8	A0A5A7YKY6	A0A429DPU7	A0A6F8YA04	A0A1Q5SFT3	A0A1E4I8V0
A0A1X0KLG8	A0A3N7ZWD8	A0A0T7FNP7	A0A5A7YFQ1	A0A6B1KJ04	A0A6F8Y9X7	A0A1R1QEU3	A0A073AZW7
A0A1X0KMF4	A0A3N8BLL9	A0A0T7GJ76	A0A5A7ZW65	A0A6B1KCK1	A0A6I3ZET9	A0A1R1QEU6	A0A222VRP3
A0A1X0YF30	A0A535ES12	A0A0T7GJ66	A0A5A7ZY18	A0A0F5RA12	A0A6I3Z9L2	A0A1X3GRH3	A0A2U1F114
A0A1X0YEV9	A0A535ERV3	A0A0T7GJD9	A0A5C1QZG1	A0A1N6ZUN2	A0A6I4M671	A0A1X3GQR9	A0A4Q7UW68
A0A1X1QWW6	A0A6J5KBQ2	A0A0X8CND5	A0A5C1R304	A0A807X012	A0A6I4M635	A0A1X3FYM5	A0A4R1HXS2
A0A1X1QWW4	A0A6J5KCZ4	A0A0X8CP41	A0A5C7WXF2	A0A2R5EZ81	A0A6N4VGZ7	A0A1X3FXS3	A0A4R6UY69
A0A1X1TB83	A0A7I8BLE5	A0A109JH28	A0A5C7Y5Z6	A0A0M1P0M7	A0A6N4VF84	A0A1X7EKJ1	A0A7Y0PQ06
A0A1X1TB85	A0A7I8BLR8	A0A120FJD5	A0A5C7Y766	A0A0Q7K7N2	A0A6N4VPS2	A0A1X7EKD0	A0A7Y9E1K5
A0A1X1WJE5	A0A850D2B2	A0A127EVA0	A0A5N0IZT1	A0A0Q9L6Q2	A0A6N4VN11	A0A1X7GMQ0	A0A897E0G9
A0A1X1XFY5	A0A850D3C1	A0A150UH78	A0A5N0IU65	A0A0Q9L817	A0A6V8KF37	A0A1X7GP25	A0A0A5FVT5
A0A1X1XFR6	B2JRQ6	A0A150UH35	A0A5N5V080	A0A0Q9NAB4	A0A6V8KP17	A0A1X7GN01	A0A024P5A3
A0A0F5NKR5	B2JRQ7	A0A150Z1P8	A0A5N5V3K3	A0A0Q9RJY1	A0A7D6IR37	A0A1Y2JGI1	A0A0B0IFK3
A0A1X1YSU0	K0DQW0	A0A150Z1Z9	A0A5S9R515	A0A0U2WN93	A0A7D6IL74	A0A1Y2JGI0	A0A1G8FTX2
A0A1X2CAH0	K0DSH0	A0A151FLU3	A0A5S9R269	A0A163KIH5	A0A7G1KJD7	A0A1Y6KIS4	A0A1G8SNL5
A0A1X2CA93	A0A1H0BMQ0	A0A151FLR0	A0A6H0S838	A0A172THL5	A0A7G1KI23	A0A1Y6KJN8	A0A1H0HHT7
A0A1X2EDV0	A0A0U2UPD5	A0A160UB13	A0A6H0SGN1	A0A1B2E935	A0A7I7JR67	A0A257PVA2	A0A1H3ZHH3
A0A1X2EEY9	A0A0V8JG18	A0A161K389	A0A6N4UYN2	A0A1H6A7I5	A0A7I7JP13	A0A257PUM4	A0A1H9KQR5

A0A386U9B7	A0A2W1NXB6	A0A160UT58	A0A6N4V2R2	A0A1H7SK12	A0A7I7JWM3	A0A2A2VM30	A0A1I3UC25
A0A386U9T4	A0A3S0W4Y7	A0A160UP78	A0A6S6PDS5	A0A1K1LLX4	A0A7I7JX36	A0A2A2VM60	A0A1I4P654
A0A3S0RKR1	A0A433HWX1	A0A160V3L5	A0A6S6P7D6	A0A1K1NFD5	A0A7I7KK81	A0A2A6NAU1	A0A1I4Q4N9
A0A3S0TKI5	A0A511VB08	A0A160V740	A0A7G8PBP4	A0A1R1B2J3	A0A7I7KIL5	A0A2A6NB65	A0A1I5L204
A0A498Q877	A0A6P1AXU8	A0A176YSA9	A0A1G4V4E3	A0A1R1C2V3	A0A7I7KXX0	A0A2A6PMY7	A0A1W5ZZI2
A0A498Q9J9	A0A1Q4AAK9	A0A176YSK9	A0A7I7MCR8	A0A1R1CFR3	A0A7I7KYL0	A0A2A6PMA2	A0A2P8H8M0
A0A4R5WTL0	A0A1Q4AAL1	A0A176Z8K7	A0A7I7MB76	A0A1R1D9L0	A0A7I7P192	A0A2D7FG24	A0A2T4U9L7
A0A7D6HMM4	A0A1H4MN71	A0A176Z6P9	A0A7I7N504	A0A1S2F692	A0A7I7P4I4	A0A2D7FFW6	A0A3N5C274
A0A7D6HS15	A0A1H4MNP3	A0A1B1UNL3	A0A7I7N4R1	A0A1X7H5G2	A0A7I7PAQ8	A0A2E7DFC6	A0A3R9Q7K2
A0A7I7KKI2	A0A0M4GS22	A0A1B1UNT7	A0A7I7SUV5	A0A1Y5JYK6	A0A7I7PAM0	A0A2E7DFD5	A0A410MC17
A0A7I7KLH5	A0A1H8D7K0	A0A1B1UPQ4	A0A7I7STK7	A0A1Y5KIG5	A0A7I7QE07	A0A2E7W3E4	A0A428MTT1
A0A7I7KUX7	A0A2N5MLH3	A0A1B1UPE2	A0A7I7T079	A0A1Y5KHH3	A0A7I7QE02	A0A2E7W3C5	A0A511W0P4
A0A7I7KU33	A0A372LTM1	A0A1B9YM30	A0A7I7T1B8	A0A385TRH3	A0A7I7QYF0	A0A2K8Y721	A0A514LLF3
A0A7I7LSX5	A0A3M8DLM5	A0A1B9YM81	A0A7I7U5W0	A0A2M9MNP6	A0A7I7QY49	A0A2K8Y7J9	A0A7T6Z194
A0A7I7NZL4	A0A4R2B6N8	A0A1C3V240	A0A7I7U4F0	A0A2N5N5W1	A0A7I7S6B5	A0A2M6U5S7	A0A7T6Z922
A0A7I7P038	W7L2Y2	A0A1C3V538	A0A7I7UPP5	A0A2R5EP56	A0A7I7S6C2	A0A2M6U5Q7	A0A841PQ82
A0A899QFU7	A0A0P1G3N7	A0A1C3V4S2	A0A7I7UMW3	A0A2V5K453	A0A7I7TMP9	A0A2M8RAQ7	W4Q4E6
A0A899QA49	A0A090DRE4	A0A1E3ECH5	A0A7I7W5H0	A0A2W4GKN0	A0A7I7TMX0	A0A2M8RAQ6	A0A1H1GGN9
A0A8E4PBX1	A0A090F418	A0A1E3EDU4	A0A7I7W8P0	A0A329MLD4	A0A7I7V1C9	A0A2S6MFC6	A0A1G8SFQ4
A0A8E4KWH2	A0A0A1YM86	A0A1E4LZM3	A0A7I7XTF6	A0A329MKE8	A0A7I7V098	A0A2S6MFC2	A0A235B665
A0A5N5USS4	A0A0E0UM74	A0A1E4LZL5	A0A7I7XTJ7	A0A378X930	A0A7I7WZI7	A0A2T2Q7S4	A0A345C3V6
A0A5N5USP2	A0A1A5RY49	A0A1G6K4D3	A0A7I9XXQ9	A0A3B0BFX2	A0A7I7WUQ5	A0A2T2Q7D2	A0A7T5JVM8
A0A0E2NMB6	A0A1A9F217	A0A1G6K4P6	A0A7I9XXV8	A0A3B0BLQ3	A0A7I7X521	A0A2T2QQF3	A0A7T7CBS4
A0A1G8WJN9	A0A1I5TZL3	A0A1G7BKU2	A0A7I9YW20	A0A3B0BNM1	A0A7I7X734	A0A2T2QQQ9	A0A7T6ZCC3
A0A3S1EIY3	A0A1X7A428	A0A1G7BKQ2	A0A7I9YW82	A0A3B0C0I8	A0A7I7XK95	A0A2U2CFU6	A0A1G8QKN2
A0A3S1ID42	A0A843ZZ47	A0A1G8JNB4	A0A7K1KH56	A0A3B0CHB1	A0A7I7XK80	A0A2U2CG61	A0A1I0X2E4
A0A434WN24	A0A222JYE2	A0A1G8JNR5	A0A7K1KGZ8	A0A430JI39	A0A7I7Z3F9	A0A2U3QBU1	A0A549YFA5
A0A3S1TQR4	A0A239PVJ5	A0A1H0FQ78	A0A7V3J2Q9	A0A410WW64	A0A7I7Z0T1	A0A2U3QC56	A0A419V4T0
A0A434PUR5	A0A2A3AL30	A0A1H0FQM1	A0A7V3J2K1	A0A4Q9DEM1	A0A7I9XR02	A0A2U8P2R3	A0A1E4I4J8
A0A3S1X5S0	A0A2A3BHV1	A0A1H1JG05	A0A7X6MUC0	A0A4Q9DLD6	A0A7I9XSV7	A0A2U8P2R1	A0A1E4I517
A0A3S1YT19	A0A2A3CJB9	A0A1H1JGT0	A0A7X6MT61	A0A4V3B538	A0A7I9YD20	A0A2U8Q0N4	A0A239M2G9
A0A440LYL3	A0A2A3CK67	A0A1H1JH45	A0A7Z7NCQ3	A0A5C4T6J6	A0A7I9YD09	A0A2U8Q2Q9	A0A239LZV7
A0A440NM24	A0A2A6M5Y5	A0A1H1ZXX4	A0A7Z7IP49	A0A5C4TAF0	A0A7I9Z141	A0A2U8QHW8	A0A2N3Y6T5
A0A3S3K1Y4	A0A2L0WPH1	A0A1H1ZYW0	A0A822MIJ7	A0A5C4TB70	A0A7I9Z0P5	A0A2U8QI06	A0A2N3Y6M0
A0A3S1G026	A0A3A5KLM4	A0A1H2B6P9	A0A822MM85	A0A5C4TAC0	A0A7I9ZTM3	A0A327M1W0	A0A402CM92
A0A3S1HMD3	A0A3P3F4Q9	A0A1H2B623	A0A837TBP5	A0A5C4TFM6	A0A7I9ZTG2	A0A327M1Y8	A0A402CM88
A0A3S1JS71	A0A3S9DTY9	A0A1H4WKW3	A0A837TDE1	A0A5C4TEN9	A0A7K0CZE8	A0A3N5NH22	A0A8F8SE91
A0A3S1RC35	A0A3S1JAU2	A0A1H4WMM4	A0A853MR77	A0A5C4TGR5	A0A7K0CZF0	A0A3N5NBQ4	A0A8F8SCE8
A0A3S1SQT9	A0A440JZX2	A0A1H4Y3Y6	A0A853MTW3	A0A5C4TGA8	A0A7K0DG61	A0A3N6NWK2	A0A2A3EW03
A0A435I6E8	A0A440VMR4	A0A1H4Y5W7	A0A895C453	A0A5Q2NK76	A0A7K0DJ47	A0A3N6PGN7	A0A2A3EVB1
A0A3S2GK92	A0A3S3EA71	A0A1H5ACD3	A0A895C469	A0A5R9GH02	A0A7K1FLZ3	A0A087LMS6	A0A1H5NEL7
A0A435WAX1	A0A3S3F9F4	A0A1H5ABW1	A0A895CKR3	A0A6B8RHY1	A0A7K1FM35	A0A087LMS7	A0A1H5NGP6
A0A435WDJ0	A0A3S3RMU5	A0A1H5F5E1	A0A895CJ16	A0A6H2GX07	A0A7L5MME0	A0A024KK32	A0A2V3UA19
A0A3S1ZWE7	A0A434SHP7	A0A1H5F585	A0A8B4R1Q4	A0A6L8V9V1	A0A7U5C954	A0A024KKA9	A0A2V3U8H5
A0A436D8P3	A0A434SSB9	A0A1H5F5C9	A0A8B4R2L7	A0A7G3E539	A0A7V3HYT3	A0A7D7WJE1	A0A7C9R8R5
A0A436GWS0	A0A434THX1	A0A1H5JC28	A0A8F9S5Z7	A0A7G8UIY0	A0A7V3HYB4	A0A7D7WLF8	A0A7C9R8S0
A0A3S2G2S6	A0A434U7E1	A0A1H5JCZ1	A0A8F9S576	A0A7G8UJH0	A0A7W9UJG8	F7QK79	A0A7J9YLR5
A0A439U3U6	A0A436SBK5	A0A1H5JCK3	A0R1M5	A0A7W0I452	A0A7W9UJG9	F7QK80	A0A511D8V1
A0A442Q9D9	A0A439HV80	A0A1H8NAV6	A0R1M6	A0A7X3K1E4	A0A7X6L021	A0A023XQV9	A0A7J9XTB6
A0A441YV57	A0A442FHK6	A0A1H8NAJ6	A4T831	A0A7X4YM04	A0A7X6KZV9	A0A023XRK4	A0A7J9ZED5
A0A442WFB3	A0A442JPC9	A0A1I3M5P3	A4T832	A0A848M3F4	A0A7T1F6X0	A0A0A3Y363	A0A5C4M0M4
A0A442XT87	A0A4R9WPA4	A0A1I3M6C3	E6TC60	D3EI84	A0A828TDJ2	A0A0A3Y6M2	A0A7J9XFK6
A0A442Z542	A0A4R9Z2B2	A0A1I3M990	E6TC61	F3M336	A0A839Q6J6	A0A0D1MQI0	A0A1E4I9H4
A0A527V2Q3	A0A502MJ26	A0A1I3M933	G7CJT9	F5LEN1	A0A839Q7A6	A0A0D1MQC7	A0A7V9T3V4
A0A530M5G1	A0A502XXJ2	A0A1I3UQ48	G7CJU0	G4HKD1	A0A846XGG9	A0A0D7P3B1	A0A2V6U2J6
A0A6L5ERZ7	A0A502Y1K8	A0A1I3ULY7	G8RYR6	H3SLB4	A0A846XIQ1	A0A0D7P874	A0A2V6TVM0
A0A6N7G4U9	A0A503VVC5	A0A1I4TJD8	G8RYR7	V9GBK8	A0A846XYE8	A0A0D7PLU8	A0A2V7AQP4
A0A7J9X7G0	A0A504AJL0	A0A1I4TJ19	I0RFQ6	W4AW75	A0A846XZL2	A0A0D7PIC1	A0A2V7ASW9
A0A7Y5WWJ1	A0A504EF69	A0A1I5BPR4	I0RFQ7	A0A3L7Y7P1	A0A846YJ65	A0A0D7QAM9	A0A1Q7IAG4
A0A807AVT2	A0A522RDC2	A0A1I5BQ17	I4BM26	A0A3L7Y8I6	A0A846YIQ5	A0A0D7Q752	A0A2V6PPP2
A0A8B3LUG1	A0A527UZZ1	A0A1I7MAY7	I4BM27	A0A4P5V545	A0A850PEZ6	A0A0E4BMI9	A0A2V6PVI6
V7ESU9	A0A528C7W8	A0A1I7MAW5	I7G628	A0A4P5V6N2	A0A850PJ67	A0A0E3VTE7	A0A2V6SA03
V7FE82	A0A5B6TQC2	A0A431PC78	I7FQT8	A0A259T0E0	A0A853LKZ7	A0A0R3CS28	A0A2V7DQ34
V7GTA5	A0A5B7VRG5	A0A3S0MKE9	K0VEA1	A0A1I4PII4	A0A853LPI9	A0A0R3DFT0	A0A2V7HWG4
V7HA15	A0A5Q8C8S7	A0A431RVV7	K0VAK4	A0A2E8WAJ5	A0A854I3X1	A0A0R3LC61	A0A2V6XMC9
X5P264	A0A6G6FPL3	A0A3S0HWE5	X5KRU1	A0A2N5KJ20	A0A220Y7G0	A0A0R3L3L3	A0A2V6XSZ5
X5P0R9	A0A6M7UY07	A0A3S5JEK8	X5L2A2	A0A2S8PNW0	A0A854UGZ3	A0A0R3MI33	A0A2V6XMI7
X5PXS9	A0A6M7WXF1	A0A3S5JEK4	A0A7X5U1V6	A0A3A0ARP8	A0A854USW0	A0A0R3MPJ0	A0A2V7CCQ3
X5S0X3	A0A6N8AC21	A0A410VEH9	A0A7X5U200	A0A3D4B937	A0A8D6HBV0	A0A109K1L9	A0A2V7C9J2
X6DBN7	A0A7D5EBI1	A0A410VHD2	A0A7G1I5U1	A0A3D4B7Q2	A0A8D6HCU6	A0A109K230	W4LVV3
X6FHX9	A0A7G6SNK3	A0A418V1G3	A0A1V3WU47	A0A4R5KDC7	A0A8H2JEX5	A0A160U8S3	A0A2V6Q3Q9
X6GP63	A0A7R7ANA2	A0A418V1I5	A0A2E4USY0	A0A4R5KDN4	A0A8H2JDZ0	A0A170PXD0	A0A2V6Q8A3
X6H3B3	A0A7R7BBV1	A0A3S0FEZ9	A0A2E4MTK2	A0A6C0NUM7	A1TCP1	A0A160V5C7	A0A2V6UQ04
X6H136	A0A7S8ESD3	A0A3S0I3P2	A0A3D2RQY0	A0A6G8QF66	A1TCP2	A0A160V4X6	A0A2V6ULL3
X6H7K0	A0A7S8EYQ2	A0A434RR28	A0A2E5G3J9	A0A6L7UF44	A1UJE3	A0A176YHD8	A0A2V6V788
X6HZ32	A0A7W1AWB1	A0A440WI93	A0A2D5Z0L8	A0A6N8WES1	A1UJE4	A0A176YEZ8	A0A2V6V5M5
A0A1A2N8J7	A0A8B3KXG5	A0A440WI14	A0A2E5IYX9	A0A7V8VWW2	B2HFG6	A0A1E3EX62	A0A2V6XJ90
A0A1A2N8M8	A0A8B3KUD7	A0A4E0QE42	A0A2E8Y3G4	A0A839TJL4	B2HFG7	A0A1E3EYL1	A0A2V6XFJ2
A0A439DP34	A0A8B3NQI2	A0A4Q0Q5V4	A0A6L7WZG7	A0A6J4V9U3	D5PEE5	A0A1G7KDZ8	A0A2V6YWY0
A0A439DP57	A0A8B5TYM7	A0A4Q0Q739	A0A2N0MK21	A0A1H0HZM2	D5PEE6	A0A1G7KEW3	A0A2V6YPW3
A0A0I9TGJ1	F7XDY9	A0A4Q0RR43	A0A2N0MTX1	A0A1H8LGS4	F5Z0E1	A0A1H0M268	A0A2V6ZKQ1
A0A0I9XPE9	M5FC01	A0A4Q0RNJ9	A0A432KD26	A0A1I4E8J5	F5Z0E2	A0A1H0M2G1	A0A2V6ZI63
A0A0M2ZDQ9	M5FGL7	A0A4Q0RW32	A0A2Z6E4S0	A0A3A0AC72	G4I1Y5	A0A1H1WER9	A0A2V6W576
A0A1X0CZ41	Q92YD0	A0A4Q0RU94	A0A1Y0BR22	A0A3D9SPT0	G4I1Y6	A0A1H1WEE8	A0A2V7BMJ8
A0A100ZAR3	V9VLT9	A0A4Q1V097	A0A3M6Q534	A0A3S0U927	H8IRU1	A0A1H4TRL0	A0A2V7CPA3
A0A100ZAJ3	A0A0Q8QC11	A0A4Q1UWL1	A0A562ZTD2	A0A3S3ERY6	H8IRU2	A0A1H4TRW6	A0A2V7CX01
A0A100ZQM5	A0A0U2UIR4	A0A4Q3YMY3	A0A7Y8KZ98	A0A4V2ZT05	I2A8Y9	A0A1H5F9I1	A0A2V7DVZ9
A0A100ZQN1	A0A2W1NDK1	A0A4Q3YMZ1	A0A3D5C1D1	A0A559KFP7	I2A8Z0	A0A1H5F9L4	A0A2V7FEX8
A0A117JM24	A0A0U2M6J9	A0A4Q4C542	A0A6L7X2G9	A0A5R9GEY0	J5E3Q1	A0A1H8HAS4	A0A2V7FE36

A0A101ADA4	A0A109JZ09	A0A4Q4C3Z4	A0A3M8CXV3	A0A5S4VW93	J4SCY8	A0A1H8HBX6	A0A3N5V946
A0A100X8F6	A0A257QYI5	A0A4Q7F589	A0A3M8CZ32	A0A6B1EUC6	J9WA00	A0A1I3MN93	A0A537QDE9
A0A100X893	A0A162JX17	A0A4Q7F544	A0A3N2GPX0	A0A6B1EU52	J9W785	A0A1I3MNT0	A0A537WFH1
A0A100ZNT6	A0A0N8HCM7	A0A4Q7G7B4	A0A8E1W6S5	A0A6L8ETT4	K0VND3	A0A1I3XRB6	A0A538R8J0
A0A117JDU3	A0A0S4QPM4	A0A4V2EA24	A0A3N6P0E4	A0A6L8EU56	K5B9J7	A0A1I3XRF2	A0A538R8I4
A0A124EXB8	A0A1S1R1U0	A0A4Q8R0G4	A0A3N6NVP3	A0A6L8V545	A0A3P3ZZC2	A0A1I5B1I2	A0A7V3F8I7
A0A124EXB9	A0A1S1RAU9	A0A4Q8QZB3	A0A3N6PS66	A0A7R6YD55	L0J377	A0A1I5B1K6	A0A0D6JUY7
A0A101BQT1	A0A1V2IK70	A0A4R0YEN2	A0A3N6PLK0	A0A7W0D3D1	L0J265	A0A1I7K692	A0A2P4NM72
A0A101BQV0	A0A1V2K452	A0A4V2NK89	A0A3S9B958	A0A7X3UGA6	M2ZJ16	A0A1I7K655	A0A2R6J1K2
A0A1A0NUE0	D3CUY1	A0A4R0YDF4	A0A351T3H5	A0A7X3UFV2	M3A322	A0A1L3F4T6	A0A365T3K0
A0A1A0NVU2	E3J885	A0A4R0YET6	A0A3D3UGR4	A0A7X3T6T0	Q742U3	A0A1L3F4N9	A0A371LT75
A0A1A0SEC0	A0A1A2YU38	A0A4R3TSW4	A0A4Q9FVB3	A0A7X3VLR9	Q742U2	A0A1M6ZV65	A0A498G610
A0A1A0SBQ6	A0A1A2RFM1	A0A4R3TR32	A0A401I0R9	H5YAM6	S4Z6A3	A0A1M6ZVI3	A0A7J4SQ07
A0A1A1YX83	A0A1A2RES3	A0A4R3X508	A0A841T2I6	V7F715	S4Z4J8	A0A1H5BQT6	A0A7J4UQR8
A0A1A1YX84	A0A1A2Z1J3	A0A4R3X8T7	A0A841UBT0	A0A1R1C186	U5WN00	A0A1M7BLJ6	M0HKU2
A0A1A1YXA2	A0A1A2Z307	A0A4R3X8R4	A0A402BG89	A0A1R1C163	U5WN55	A0A1M7U7S2	M0NFE5
A0A1A2L7L6	A0A1Q4I2F9	A0A4R5QE81	A0A401ZSL3	A0A1I3QS29	V7J1Z5	A0A1M7U784	A0A4C2ELN2
A0A1A2L8R9	A0A1Q4I307	A0A4R5QE79	A0A402AZC4	A0A1I3V8D6	V7IZP4	A0A1Q4BCV6	A0A1H3IHP9
A0A1A3CFK3	A0A1E3ZJK2	A0A4R5QJR8	A0A4P6K5B9	A0A1I3V8X2	V7JB23	A0A1Q4BC24	A0A1H6FXX3
A0A1A3CE13	A0A1Q3XJF4	A0A4R5QLK5	A0A4P6K743	A0A1I3V7M1	V7JD70	A0A1Q5RQ66	A0A2B7GP79
A0A1A3TB87	A0A3G8GQU3	A0A4V6BF10	A0A5A5TI09	A0A1I3V8P4	V7KAD0	A0A1Q5RQB2	A0A2R6DNC2
A0A1A3TAA8	A0A4Q7N6I7	A0A4U6BNG3	A0A424L778	A0A1I4IPS2	V7KCA1	A0A1R1QZY6	A0A365T5Z7
A0A1A3TEL5	A0A1E4QRB1	A0A4V6Y1G8	A0A1Z8RAK7	A0A327M5R1	V7KLJ3	A0A1R1R092	A0A498G1Z0
A0A1A3TG34	A0A1Y0BGX5	A0A4U6C0F3	A0A1Q7DGE1	A0A327MCD3	V7KI40	A0A1X3H9Z0	A0A7D5HZC5
A0A1E3RUM6	A0A240TPT4	A0A4U6BXI7	A0A1Q8A0S1	A0A328U1U0	V7KZ46	A0A845IPC7	D2S2D6
A0A1E3RUL6	A0A240UI30	A0A4U6RU97	A0A536K0M3	A0A495ZBN2	V7KVK6	A0A845IL49	D8JBZ1
A0A1H6KZ62	A0A4Q2IZM5	A0A4U6RW99	A0A536RGM4	A0A495ZBD7	V7L8R8	A0A2A2VIG8	L9XRM7
A0A1H6L4L9	A0A8G0UHD2	A0A4R4DTY5	A0A536GCW9	A0A4V6MSD7	V7MVS4	A0A2A2VIN4	A0A1H3EF00
A0A1H6L530	A0A1F4CB47	A0A4R4DVI8	A0A536RRC3	A0A4R5K7Z3	W5TJ21	A0A2A6NTF5	A0A1C1A9I6
A0A1X0BRP6	A0A1G7KE46	A0A4R3DV26	A0A536JIF9	A0A4R5K7A6	W5TIH8	A0A2A6NTN1	A0A1V4H6F9
A0A1X0BRS7	A0A498KTD9	A0A4R3DYP3	A0A536KPD1	A0A4V2ZSB1	W9AU56	A0A2S6NIH5	A0A1F3ZAK9
A0A1X0HGF9	A0A1G8KI22	A0A4Y9KX32	A0A536RAZ5	A0A4R5KBV8	W9ATB7	A0A2S6NIF4	A0A1Q6WFV5
A0A1X0HF44	A0A1H6CPD0	A0A4Y9KTK6	A0A536ZZL4	A0A4R5KK47	X7V456	A0A2T2QSV6	A0A1Q7EEB1
A0A1X0HEV9	A0A498GBG7	A0A4Y9LVB4	A0A535N661	A0A525KPZ0	X7V6N4	A0A2T2QSP5	A0A1Q7NUH4
A0A1X0JM47	A0A1H6B9H9	A0A4Y9LVB3	A0A535DLK1	A0A525KPQ6	X7ZHE6	A0A3C0HJ36	A0A1Q7PFP5
A0A1X0JLE9	A0A2V6PMR6	A0A4Y9M115	A0A535ZSF3	A0A559K5Z6	X7ZGH4	A0A3C0HJT5	A0A1Q7SN61
A0A2A7MRW3	A0A1T3NZT9	A0A4Y9M1D4	A0A535UPT5	A0A559K5U7	A0A7R7MQJ7	A0A410VDQ1	A0A1Q7UJV7
A0A2A7MT14	A0A401YRV8	A0A4Y9NS09	A0A3N2GPB7	A0A559K8C6	X8A3P6	A0A410VDT7	A0A1Q7XWT2
A0A2S8KAU3	A0A6I5DJ76	A0A4Y9NQ25	A0A3N2GP95	A0A559K9P0	X8CPU0	A0A4Q0RNS8	A0A2V6X621
A0A2S8K9U8	A0A7K2YJT0	A0A4Y9UAW2	A0A510Y755	A0A559KFY5	X8CN27	A0A4Q0RL91	A0A2V6YVV3
A0A318HK58	A0A1V4TYW2	A0A4Y9UFG2	A0A1H2V7R7	A0A7V9SCU7	A0A0E2WG64	A0A4Q1UG16	A0A2V7A902
A0A318HK68	A0A1V4TZ09	A0A4Y9UMT5	A0A5Q0TPL6	A0A7X1B1V5	A0A0E2WA50	A0A4Q1UIW4	A0A2V6VST4
A0A370D093	A0A7J4N7Y3	A0A4Y9UKQ0	A0A5Q0TSS5	A0A7X5C1G2	A0A0F5NIU3	A0A4V1WED1	A0A2V7BLA1
A0A370D291	A0A7L4QIS3	A0A508T4Q6	A0A8F5BPA3	A0A831TSR9	A0A0F5NIY5	A0A4Y3Z7A0	A0A2V7DSZ0
A0A537JY73	A0A843B797	A0A508SYN8	A0A8F5GTI5	A0A831TP66	A0A1X1U989	A0A4U6S8Y1	A0A2V7E3B4
A0A537JYB0	A0A257Q1T4	A0A508TYX3	A0A653N5E3	A0A849MUH7	A0A1X1VS20	A0A4U6SF32	A0A2V6Z1Q9
A0A7I7UQH8	A0A090MMQ1	A0A508TYY4	A0A2N2VF94	A0A849MVV4	A0A1X1VS70	A0A4Y9LUU3	A0A2V6TQ36
A0A7I7UST9	A0A1M5WA98	A0A516J016	N6Y0X9	A0A8A0DIT7	A0A1X1W3J5	A0A4Y9LUS7	A0A2V6VC26
A0A7I7UWZ6	A0A380WCA3	A0A516IZW1	A0A6A8N0H3	A0A845CDQ2	A0A1X1W3N5	A0A4Y9UWS2	A0A2V7A3Q1
A0A7I7UX86	A0A431NWW5	A0A525J7T6	A0A6J6P0Q7	E7QZK8	A0A1X1YS73	A0A4Y9UWP9	A0A2V7AEF5
A0A7I7WHK9	A0A3S3AZT6	A0A525J7H9	A0A6G0EC42	A0A1D2QC20	A0A1X1YS44	A0A508T2T1	A0A2V7BPJ2
A0A7I7WKJ3	A0A4Q0MCN8	A0A525K4N9	A0A6A8GJX8	A0A1H8MUA7	A0A1X1YVJ9	A0A508SZC1	A0A2V7C1N1
A0A7J9YJD7	A0A522GG73	A0A525K4N6	A0A0A1YRF6	A0A285P6T1	A0A1X1YVJ8	A0A560EAF4	A0A2V7CX80
A0A8G1AZN3	A0A525KAT6	A0A533J617	A0A220SN51	A0A2P4NMU3	A0A1X1ZE64	A0A560EAE8	A0A2V7FAN3
A0A8G1B1S9	A0A7W6D1N3	A0A533J616	A0A2Z5TZZ9	A0A4Q9DRG3	A0A1X1ZDZ3	A0A560JZ75	A0A7J9X2I3
G8RRB9	B6JH23	A0A537LEP5	A0A359DC54	A0A4U7F3N1	A0A1X1ZZE2	A0A560JZL3	A0A7J9WPR7
G8RRC0	D6V8E7	A0A537LFC5	A0A3M6Q364	A0A5P9P2G3	A0A1X1ZZL1	A0A560M0S0	A0A7K0A5N1
L0IXX0	A0A2A5XEX9	A0A537M5N5	G6YS19	A0A5P9P9D7	A0A1X2A3I5	A0A560LYA0	A0A7J9WHI4
A0A7D4Y4U9	A0A1X2A3V2	A0A562RDP9	A0A7K0A4P3	A0A2E5IX63			

Table S5. Uniprot IDs of candidate AROs that contains small PF00355 protein within their genome neighborhood.

Supplementary References

(1) J. A. Gerlt, *et al.*, Enzyme Function Initiative-Enzyme Similarity Tool (EFI-EST): A web tool for generating protein sequence similarity networks. *Biochim. Biophys. Acta - Proteins Proteomics* **1854**, 1019–1037 (2015). https://doi.org/10.1016/j.bbapap.2015.04.015.
(2) P. Shannon, *et al.*, Cytoscape: A Software Environment for Integrated Models of Biomolecular Interaction Networks. *Genome Res.* **13**, 2498–2504 (2003). https://doi.org/10.1101/gr.1239303.
(3) W. Kabsch, XDS. *Acta Crystallogr. Sect. D Biol. Crystallogr.* **66**, 125–132 (2010). https://doi.org/10.1107/S0907444909047337.
(4) W. Kabsch, The rotation method in crystallography edited by U. W. Arndt and A. J. Wonacott. *Acta Crystallogr. Sect. B Struct. Crystallogr. Cryst. Chem.* **34**, 1049–1050 (1978). https://doi.org/10.1107/S0567740878004896.
(5) M. D. Winn, *et al.*, Overview of the CCP 4 suite and current developments. *Acta Crystallogr. Sect. D Biol. Crystallogr.* **67**, 235–242 (2011). https://doi.org/10.1107/S0907444910045749.
(6) A. Waterhouse, *et al.*, SWISS-MODEL: homology modelling of protein structures and complexes. *Nucleic Acids Res.* **46**, W296–W303 (2018). https://doi.org/10.1093/nar/gky427.
(7) E. F. Pettersen, *et al.*, UCSF ChimeraX: Structure visualization for researchers, educators, and developers. *Protein Sci.* **30**, 70–82 (2021). https://doi.org/10.1002/pro.3943.
(8) A. B. Roy, M. J. E. Hewlins, Sulfoquinovose and its aldonic acid: their preparation and oxidation to 2-sulfoacetaldehyde by periodate. *Carbohydr. Res.* **302**, 113–117 (1997). https://doi.org/10.1016/S0008-6215(97)00112-2.
(9) S. Stoll, A. Schweiger, EasySpin, a comprehensive software package for spectral simulation and analysis in EPR. *J. Magn. Reson.* **178**, 42–55 (2006). https://doi.org/10.1016/j.jmr.2005.08.013.
(10) E. Eichhorn, J. R. Van Der Ploeg, M. A. Kertesz, T. Leisinger, Characterization of α-ketoglutarate-dependent taurine dioxygenase from Escherichia coli. *J. Biol. Chem.* **272**, 23031–23036 (1997). https://doi.org/10.1074/jbc.272.37.23031.
(11) A. Matthews, *et al.*, Bacterial flavoprotein monooxygenase YxeK salvages toxic S ‐ (2 ‐ succino) ‐ adducts via oxygenolytic C ‐ S bond cleavage. *FEBS J.* **289**, 787–807 (2022). https://doi.org/10.1111/febs.16193.
(12) M. Alcalde, E. T. Farinas, F. H. Arnold, Colorimetric high-throughput assay for alkene epoxidation catalyzed by cytochrome P450 BM-3 variant 139-3. *J. Biomol. Screen*. **9**, 141–146 (2004). https://doi.org/10.1177/1087057103261913.
(13) T. D. Schneider, R. M. Stephens, Sequence logos: a new way to display consensus sequences. *Nucleic Acids Res*. **18**, 6097–6100 (1990). https://doi.org/10.1093/nar/18.20.6097.
